# Transcriptome Analysis of Environmental *Pseudomonas* Isolates Reveals Mechanisms of Biodegradation of Naphthenic Acid Fraction Compounds (NAFCs) in Oil Sands Tailings

**DOI:** 10.3390/microorganisms9102124

**Published:** 2021-10-09

**Authors:** Parisa Chegounian, Stephane Flibotte, Kerry Peru, John Headley, Dena McMartin, Bryne Gramlich, Vikramaditya G. Yadav

**Affiliations:** 1 Department of Chemical and Biological Engineering, The University of British Columbia, Vancouver, BC V6T 1Z3, Canada; parisach@mail.ubc.ca; 2 UBC/LSI Bioinformatics Facility, University of British Columbia, Vancouver, BC V6T 1Z3, Canada; stephane.flibotte@ubc.ca; 3 Watershed Hydrology and Ecology Research Division, Water and Science Technology Directorate, Environment & Climate Change Canada, Saskatoon, SK S7N 3H5, Canada; kerry.peru@canada.ca (K.P.); John.Headley@canada.ca (J.H.); 4 Department of Civil, Geological and Environmental Engineering, University of Saskatchewan, Saskatoon, SK S7N 5A9, Canada; dena.mcmartin@usask.ca; 5 Allonnia L.L.C., Boston, MA 02210, USA; bgramlich@allonnia.com; 6 School of Biomedical Engineering, The University of British Columbia, Vancouver, BC V6T 1Z3, Canada

**Keywords:** naphthenic acids, pseudomonads, biodegradation, transcriptomics, bioinformatics, microbial consortia

## Abstract

Naphthenic acid fraction compounds (NAFCs) are highly recalcitrant constituents of oil sands tailings. Although some microorganisms in the tailings can individually and synergistically metabolize NAFCs, the biochemical mechanisms that underpin these processes are hitherto unknown. To this end, we isolated two microorganisms, *Pseudomonas protegens* and *Pseudomonas putida*, from oils sands tailings and analyzed their transcriptomes to shed light on the metabolic processes employed by them to degrade and detoxify NAFCs. We identified 1048, 521 and 1434 genes that are upregulated in *P. protegens*, *P. putida* and a 1:1 co-culture of the strains, respectively. We subsequently enumerated the biochemical activities of enriched genes and gene products to reveal the identities of the enzymes that are associated with NAFC degradation. Separately, we analyzed the NAFCs that are degraded by the two pseudomonads and their 1:1 co-culture and determined the composition of the molecules using mass spectrometry. We then compared these molecular formulas to those of the cognate substrates of the enriched enzymes to chart the metabolic network and understand the mechanisms of degradation that are employed by the microbial cultures. Not only does the consortium behave differently than the pure cultures, but our analysis also revealed the mechanisms responsible for accelerated rate of degradation of NAFCs by the co-culture. Our findings provide new directions for engineering or evolving microorganisms and their consortia for degrading NAFCs more stably and aggressively.

## 1. Introduction

The oil sands in the Athabasca region of northern Alberta is an unconventional and plentiful energy reserve [1]. The mining and extraction of bitumen from these deposits consumes copious quantities of water. Conversely, bitumen extraction also generates a large volume of wastewater called oil sands process-affected water (OSPW) [2]. This waste stream is chronically and acutely toxic to aquatic life [3,4,5,6,7], and the principal toxicants in OSPW are a large group of organic molecules called naphthenic acids fraction compounds (NAFCs). The general formula of NAFCs is C_c_H_h_N_n_O_o_S_s_ and their carbon content ranges between seven and 26 atoms [8,9,10]. OSPW contains several hundred NAFCs that can be further divided into O_o_, N_n_O_o_, O_o_S_s_, N_n_S_s_ and N_n_O_o_S_s_ compounds [5,11]. Several of these molecules are unsaturated, and the degree of unsaturation of an NAFC can be inferred from its double bound equivalents (DBE) [12,13], which is estimated as c + ½(n − h) + 1. Most of the debate surrounding the toxicity of OSPW in the popular press, however, has centered on a specific sub-group of NAFCs known as classic naphthenic acids (NAs). These molecules have an empirical formula of C_c_H_h_O_2_ and have been determined by environmental researchers to be the most toxic NAFCs [14,15,16]. Current environmental regulations forbid direct discharge into surrounding bodies of water [17,18], and the OSPW is stored on-site in large tailings ponds while it awaits detoxification. However, NAFCs are notoriously recalcitrant, which makes OSPW very difficult to treat effectively and economically. As a consequence, the volume of untreated OSPW in the tailings ponds is continually rising and the inventory of water now exceeds 700 billion liters. This large volume of toxic water has amplified concerns about seepage into bodies of ground and surface water and contamination of sources of drinking water, not to mention the risks of a catastrophic spill. There is an unmet, urgent need for the development of wastewater treatment technologies that can economically rehabilitate OSPW [19].

The high concentration of NAFCs and other toxic compounds in the OSPW tailings ponds has sustained a unique ecological niche that abounds with microorganisms that have evolved to sense, uptake and metabolize these compounds and survive in this extreme environment [20,21,22]. *Proteobacteria* is the most abundant phylum in the microbiome in OSPW tailings ponds [14,23,24], and, herein, several microbial species of the genus *Pseudomonas* have been shown to degrade a subset of NAs and detoxify OSPW, albeit at exceedingly slow rates [16,25,26,27,28,29]. Microbial bioremediation clearly is a promising solution for decontamination and detoxification of OSPW. However, the range of structures and the rates at which NAFCs are degraded by these native microorganisms necessitates significant improvements in order for bioremediation to be sustainable and economical at scales required by oil sands operators [30,31,32]. Methodologies such as targeted genome engineering and adaptive laboratory evolution will be central to improving the substrate range and rate of biodegradation of NAFCs but these techniques require sound understanding of the biochemical pathways involved [32]. Unfortunately, the mechanisms of biodegradation of NAFCs are either poorly understood or have been delineated for idealized substrates [29,33,34,35,36,37,38,39,40,41]. In fact, the biodegradation pathways of most NAFCs in OSPW, notably N_n_O_o_, O_o_S_s_, N_n_S_s_, and N_n_O_o_S_s_ compounds, are entirely unknown. Elucidation of the genes, enzymes and metabolic pathways responsible for adaptation to and degradation of NAFCs is necessary in order to effectively and economically remediate OSPW.

The first step in investigating the mechanisms of biodegradation of NAFCs is isolating and enriching appropriate microbial chassis that could serve as key constituents of the bioremediation platform. Next, analyzing the transcriptomic responses using RNA-seq of the microorganisms to exposure to NAFCs sheds light on the genes, enzymes and metabolic pathways responsible for adaptation to and degradation of the toxic compounds [42,43]. We previously isolated two pseudomonads, *P. protegens* and *P. putida*, that can metabolize NAFCs in OSPW by exploiting their ability to grow on these compounds as the sole source of carbon in plate and liquid cultures [44]. Chemical analyses of the culture medium before and after biological activity revealed that the two microorganisms exhibit unique propensities and rates for degrading NAFCs and that these differences are more pronounced for classic NAs. In the current study, we have employed RNA-seq to elucidate the mechanisms of biodegradation of NAFCs by individual and co-cultures of *P. protegens* and *P. putida*. Till date, no studies have been performed on transcriptomic response of microorganisms isolated from OSPW tailings ponds. In addition to deducing the transcriptional responses of *P. protegens* and *P. putida*, we also correlated this information to mass spectrometry data to identify NAFCs as substrates of individual enzymes. The substrates were then classified based on their heteroatom compositions and the enzyme-substrate pairs were then used to construct the biodegradation pathways. Finally, we also compared the metabolic networks of the two strains in individual and 1:1 co-culture with an eye on developing consortia for biodegradation.

## 2. Materials and Methods

### 2.1. Extraction and Characterization of NAFCs

OSPW samples were kindly provided by Suncor Energy. All chemicals and consumables used in this study were purchased from Fisher Scientific Canada (Ottawa, ON). We stored the samples in sealed polyethylene containers in the dark at 4 °C. To test the samples, we first removed suspended particulate matter from 1 L batches of OSPW using vacuum filtration through grade-4 glass fiber filters having a nominal particle retention size of 1.2 μm. The pH of the filtered water was then lowered to pH 2 using H_2_SO_4_ and NAFCs were subsequently extracted with 100 mL of dichloromethane (DCM). The OSPW was treated twice with the same batch of DCM to maximize the concentration of the extracted NAFCs. For analytical characterization, the organic fraction was dissolved in a 1:1 solution (by volume) of acetonitrile and deionized water until the nominal concentration of NAFCs in the solution matched that of the original OSPW sample. We analyzed this mixture using HPLC-Orbitrap mass spectrometry. Details about the chromatography and mass spectrometry methods have been detailed in the accompanying Electronic Supporting Information (ESI) package.

### 2.2. Genome Sequencing and Assembly

**Figure 1:**
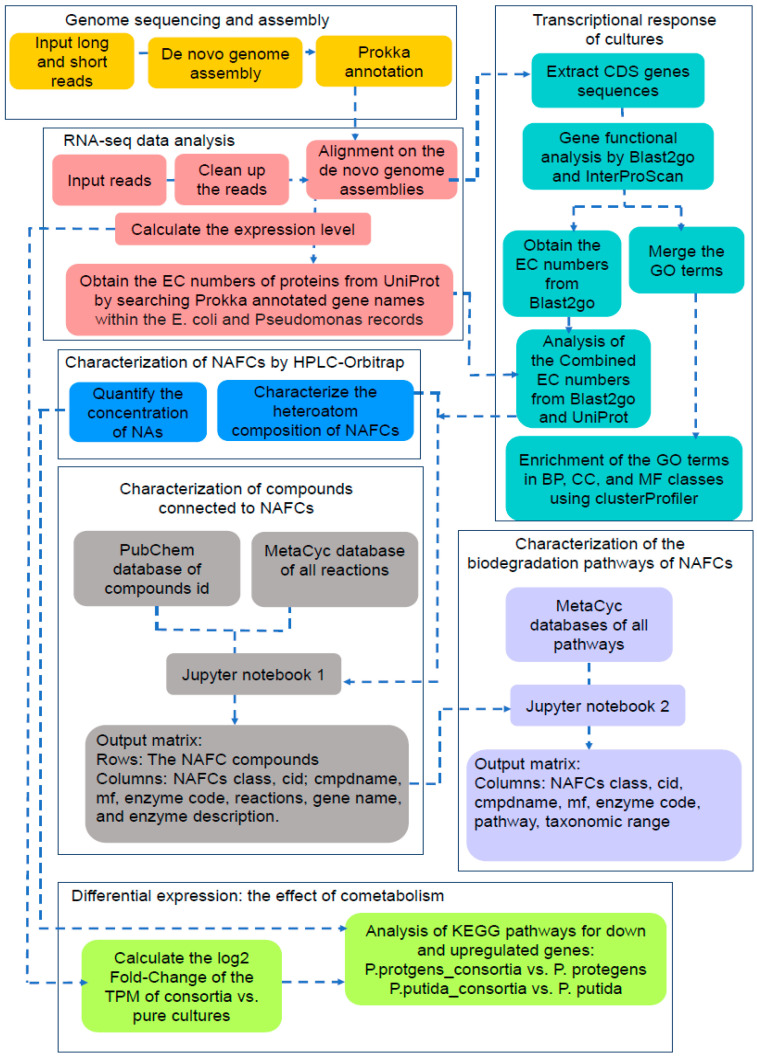
Data analysis workflow. Genome sequencing and assembly use long and short reads as inputs (**yellow boxes**). Short reads in the FastQ files of the RNA-seq libraries of pure *P. putida*, *P. protegens* and the 1:1 co-culture are aligned to the annotated genomes (**red boxes**). These alignments later serve as starting points for analyzing the transcriptional response of the microorganisms in either the pure cultures or co-culture by correlating chemical data (**blue boxes**) with GO terms and Enzyme Commission numbers (**teal boxes**). Cheminformatic analyses of the degraded NAFCs (**grey boxes**), which includes further categorization of the molecules by their class, PubChem ID (id), name (cmpdname) and molecular formula (mf), allows comparisons between the structures of the NAFCs and those of canonical substrates of enzymes determined previously (**blue boxes**). This step yields further information about the enzymatic reactions and their genes, which, in turn, reveals the biodegradation pathway (**purple boxes**). We also calculated fold-changes of the overexpressed and down-regulated genes (**lime boxes**) to provide insights about co-operative metabolism by the strains in the co-culture.

We extracted high-molecular weight DNA from the water samples using chemical extraction. We verified the fragment size distribution, quantified the concentration of DNA and sequenced the libraries using the Oxford Nanopore and Illumina platforms. The methodology used for the genome assembly and the statistics (Table S1) have been summarized in the accompanying ESI package. We then used the Prokka package to predict and annotate the genes (Figure 1, yellow boxes) [45].

### 2.3. Evaluating Biodegradation of NAFCs by the Microbial Cultures

We once again filtered the OSPW using grade-4 glass fiber filters having a nominal particle retention size of 1.2 μm to eliminate particulate matter. Separately, we prepared starter cultures of *P. putida* and *P. protegens* by directly inoculating LB medium with glycerol stocks of the strains. The two cultures were propagated overnight at 22 °C under agitation at 200 rpm and were then used to separately inoculate 50 mL of filtered OSPW in 250 mL shaker flasks. In addition to pure cultures of the two strains, we also prepared a 1:1 co-culture. All cultures had a starting optical density (OD_600_) of 0.1. The three cultures were then propagated for 30 days at 22 °C under constant agitation at 200 rpm. The extent of degradation of NAs by the cultures over the 30-day period was then quantified using HPLC-Orbitrap mass spectrometry. Additionally, we also resuspended the extracted NAFCs in deionized water to a level that matches their original concentration in OSPW. We subsequently assessed the toxicity (EC50) of the resuspended NAFC solution on *Vibrio fischeri* by quantifying the inhibition of growth over a 15 min duration [46].

### 2.4. RNA Extraction, Library Preparation and Sequencing

Three tester samples of M9 media supplemented with 280 mg/L of the extracted NAFCs were inoculated with *P. putida*, *P. protegens* and a 1:1 co-culture of the two strains. The starting OD_600_ in each culture was 0.1. Driver samples were identically prepared, albeit without a carbon source. Suppression subtractive hybridization (SSH) was then employed to prepare libraries of transcripts associated with up-regulated genes in the tester samples compared to the corresponding driver samples [47]. Total RNA was isolated from all six testers and drivers using a PureLink^TM^ RNA Mini Kit (Thermo Fisher Scientific, Ottawa, ON, Canada) by following the manufacturer’s protocol. Subsequently, 2 μg of RNA per sample was used to synthesize cDNA, amplify the up-regulated transcripts by SSH, and generate RNA libraries using the PCR-Select™ cDNA Subtraction Kit (Takara Bio, San Jose, CA, USA). RNA-seq libraries were prepared thereafter and sequenced on the Illumina HiSeq platform using a paired protocol of 2 × 150 bp (Genewiz, Seattle, WA, USA). A cosmic summary of the sequencing results of the RNA-seq libraries has been provided in Table S2 in the ESI package.

### 2.5. Analysis of the RNA-seq Data

The reads were aligned to our de novo genome assemblies with the BWA tool [48]. The resulting bam files were sorted by coordinates and PCR duplicates were removed from the sorted reads using SAMtools. On the other hand, the RNA-seq library generated from the co-culture was aligned to a composite genome comprising of the genomes of *P. putida* and *P. protegens*. Read counts at the gene levels were then estimated for each RNA-seq library using the bedtools toolkit [49]. We then calculated the Transcripts Per Million (TPM) values using an in-house Perl script. We only retained genes that were covered by sequencing reads for at least 150 bases or 90% of their length (Figure 1, red boxes) for the subsequent analyses.

### 2.6. Characterization of the Genes Involved in Biodegradation of NAFCs

We characterized the transcriptional responses of the three cultures by determining the Gene Ontology (GO) annotations and Enzyme Commission (EC) numbers of the gene products that were previously identified using RNA-seq (Figure 1, teal boxes). Functional annotation of the genes for each RNA-seq library was performed using GO annotations with Blast2GO, InterProScan [50] and the nr database of the *Pseudomonas* genus with a BLAST expectation value of 1 × 10^−3^. The GO terms from Blast2GO and InterProScan were then merged and used for characterization of the transcriptional responses of the *Pseudomonas* cultures to NAFCs. The statistical over-representation analysis for GO terms was performed using the universal enricher function of the clusterProfiler package from Bioconductor [51]. We focused on the biological processes (BPs) and molecular functions (MFs) clusters owing to the high likelihood of these genes being involved in biodegradation of NAFCs. Similarly, we assessed the Enzyme Commission (EC) numbers by cross-referencing the results of Blast2GO’s EC mapping function with EC numbers of the products of *E. coli* and *Pseudomonas* spp. genes in the UniProt database that were annotated by Prokka. In order to determine the metabolic network responsible for biodegradation of NAFCs, we developed an entirely novel approach that co-analyses transcript and chemical data. The code for the analysis is included in the two Jupyter notebooks in the ESI package.

Briefly, the code in the first notebook matches the EC numbers of gene products identified from the RNA-seq libraries with enzymatic reactions in the MetaCyc database, fetches the Pubchem IDs of the substrates for the identified reactions and then retrieves their molecular formulas from the Pubchem database. Next, the algorithm classifies the retrieved molecular formulas into NAFC groups based on their heteroatom composition. These groups include O_o_, N_n_O_o_, O_o_S_s_, S_s_, N_n_, N_n_O_o_S_s_ and CH NAFCs. Differences between the substrates deduced for the pure cultures and co-cultures were assessed statistically with the aid of the Pearson’s Chi square test for count data in R using the function chisq.test. The compounds identified using the aforementioned algorithm were subsequently inputted to the second Jupyter notebook. Due to over-generality of the integrated pathway maps on the Kyoto Encyclopedia of Genes and Genomes (KEGG) pathway database, we used more localized and individualized maps on MetaCyc to identify the NAFC degradation pathways. The algorithm finds and matches the chemical IDs (CID) of the substrates to EC numbers of putative enzymes, and later interrogates all combinations of CID-EC combinations in the entire MetaCyc database. This search outputs two data frames for the CID-EC pairs, one with and the other without pathway information. These data frames are re-classified into the dominant NAFC groups (e.g., O, N, NO, NOS, S and SO compounds, among others), which finally allows for the determination of the presence or absence of CID-EC pairs in specific pathways. We also investigated the effect of co-operative metabolism in the co-culture by calculating the fold-change of the TPM values for *P. putida* and *P. putida* when the strains are cultured individually or together. Specifically, we identified KEGG pathways in *P. putida* and *P. protegens* whose constituent enzymes exhibited fold-changes lower and greater than 1 in the pure cultures or co-culture.

## 3. Results

### 3.1. Biodegradation of NAFCs by the Pseudomonas Cultures

**Figure 2:**
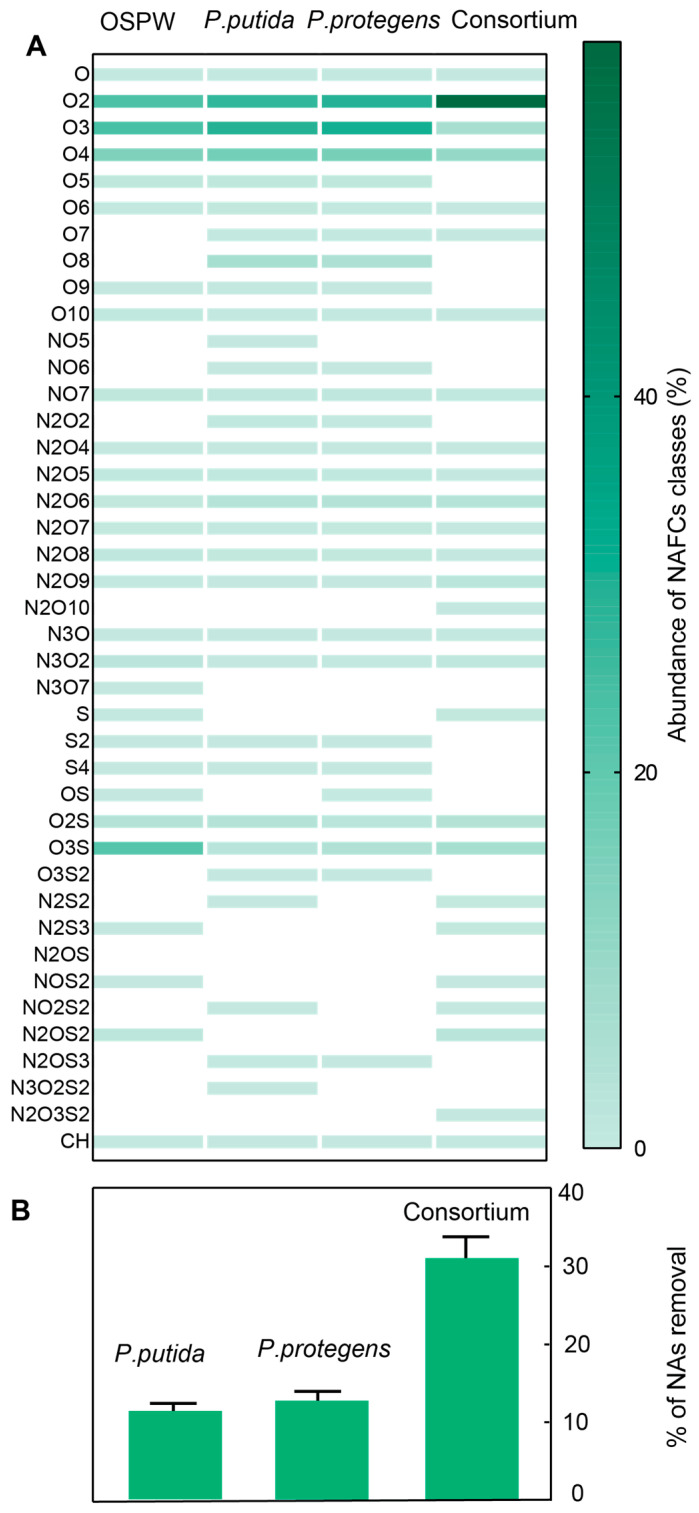
HPLC-Orbitrap results. (**A**) We quantified the relative abundance of the different classes NAFCs in OSPW before and after degradation by pure cultures and a 1:1 co-culture of *P. putida* and *P. protegens.* (**B**) We also estimated the total amount of NAs removed by the three cultures. NAs are the major toxicants in OSPW.

We quantified the substrate range (Figure 2A) and extent (Figure 2B) of biodegradation of NAFCs in OSPW by pure cultures and a 1:1 co-culture of *P. putida* and *P. protegens* after a 30-day treatment. OSPW and all three microbially-treated water samples contain a significantly higher fraction of O_2_, O_3_ and O_4_ NAFCs compared to other oxygenated species. Additionally, with the exceptions of SO_2_ and SO_3_ species, the proportion of S- and N-containing NAFCs is also relatively low in all four samples. This chemical distribution is consistent with previous reports. Additionally, O_7_, O_8_, N_2_O_2_, and S_2_O_3_ groups do not occur in OSPW but are generated by microbial metabolism.

On the other hand, S, N_2_S_3_, NOS_2_ and N_2_OS_2_ NAFCs are completely removed from OSPW after biodegradation. We speculate that these compounds are consumed by microorganisms as a source of sulfur. We also quantified the concentration of classic NAs in OSPW and their extent of removal by microbial activity. NAs are the major toxicants in OSPW [5]. We identified as many as 131 unique compounds that had a cumulative concentration of 28.2 mg/L. Their carbon numbers and DBEs ranged between 5–25 and 1–10, respectively. Of these, species containing 16 carbon atoms comprised 20.1% of the population of classic NAs, whereas compounds with a DBE of 4 accounted for 21.3% of the population. Aliphatic compounds most commonly have a DBE of 1, whereas the DBEs of alicyclic compounds range between 2–4. The raw gas chromatograms revealed that aliphatic and alicyclic NAs exhibit longer column retention times compared to aromatic and polycyclic compounds in our experiment. The pure cultures of *P. putida*, *P. protegens* and the 1:1 co-culture reduced the concentration of NAs in the culture media by 11%, 12% and 31%, respectively (Figure 2B). These extents of degradation are roughly twice that of values detected by other groups, albeit using different, possibly niche *Pseudomonas* isolates [28]. We also plotted the difference between the concentrations of an individual NAFCs before (C_io_) and after (C_i_) microbial treatment as a heat map for each of the three cultures (Figure S1 in the ESI package).

The plot confirms that the degradation profiles of the three cultures is distinct, which has implications for designing consortia. Additionally, we also observed that the concentrations of some NAFCs increased after microbial treatment, which reveals these species to be products of the degradation of other NAFCs. Finally, while the resuspended solution of extracted NAFCs elicited an average EC50 value of 82%, treatment with pure cultures of *P. putida, P. protegens* and the 1:1 co-culture reduced the toxicity of the solution to EC50 values of 94%, 92% and 100%, respectively.

### 3.2. Transcriptional Responses of Pure Cultures of the Pseudomonas Isolates to NAFCs

SSH is a simple and effective technique for generating cDNA that is highly enriched in differentially expressed genes that are present in either high or low abundance. The methodology permits simultaneous normalization and subtraction, which facilitates identification of over-expressed genes even for rare transcripts. The latter is critical for identification of novel genes that are selectively expressed upon exposure to NAFCs. The combination of high levels of enrichment, low background and normalized abundance of cDNA in the subtracted mixtures is ideal for rapid cloning of cDNA of the differentially expressed genes. Exposure to NAFCs elicited the expression of 1048 and 521 genes in pure cultures of *P. protegens* and *P. putida*, respectively. A total of 1434 genes were differentially expressed by cells in the 1:1 co-culture, of which 993 genes were expressed by *P. protegens*. It is notable that *P. protegens* induces many more genes than *P. putida* either separately or in co-cultures.

**Figure 3:**
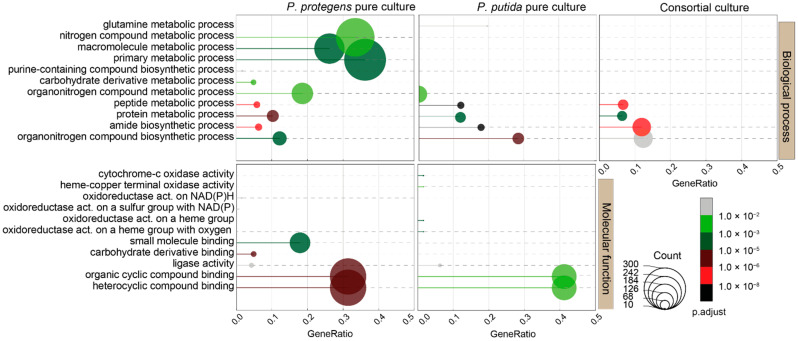
Over-representation of GO terms in pure and co-cultures of *P. protegens* and *P. putida*. We obtained enriched GO terms using Blast2GO, InterProScan and the nr database of the *Pseudomonas* genus with a BLAST expectation value of 1 × 10^−3^, and then merged these results to calculate the GeneRatios (number of genes that over-expressed compared to the number of genes with the same term in the whole genome), adjusted p-values and the number of genes that were enriched in the cultures.

Although GO terms for cellular component (CC), biological process (BP), and molecular function (MF) were statistically over-represented (P-adj < 0.05) in all the cultures, we only considered the BP and MF classes for further analyses owing to the greater likelihood of their involvement in the biodegradation of NAFCs. We have summarized the enriched GO terms in Table S3 in the ESI. In total, 11 GO terms were significantly over-represented in the BP class (Figure 3), of which 4 GO terms, namely organonitrogen compound biosynthetic process (GO:1901566), amide biosynthetic process (GO:0043604), protein metabolic process (GO:0019538) and peptide metabolic process (GO:0006518) were common to all the cultures. Five GO terms corresponding to carbohydrate derivative metabolic process (GO:1901135), purine-containing compound biosynthetic process (GO:0072522), primary metabolic process (GO:0044238), macromolecule metabolic process (GO:0043170) and nitrogen compound metabolic process (GO:0006807) were uniquely enriched in the cultures of *P. protegens*. On the other hand, glutamine metabolic process (GO:0006541) was the only unique GO term that was enriched in cultures of *P. putida*. The GO terms for organonitrogen compound metabolic process (GO:1901564) was only biological process that was uniquely over-represented in both pure cultures. Similarly, GO terms in the MF class corresponding to heterocyclic compound binding (GO:1901363), organic cyclic compound binding (GO:0097159) and ligase activity (GO:0016874) were enriched in both pure cultures but no terms were uniquely enriched in the 1:1 co-culture.

The pure cultures of *P. protegens* uniquely exhibited GO terms for carbohydrate derivative binding (GO:0097367), small molecule binding (GO:0036094), oxidoreductase activity on a sulfur group of donors with NAD(P) as acceptor (GO:0016668) and oxidoreductase activity acting on NAD(P)H (GO:0016651). GO terms corresponding to oxidoreductase activity on a heme group of donors with oxygen as acceptor (GO:0016676), oxidoreductase activity on a heme group of donors (GO:0016675), heme-copper terminal oxidase activity (GO:0015002), and cytochrome-c oxidase activity (GO:0004129) were uniquely enriched in pure cultures of *P. putida*.

We consolidated the results from Blast2GO and UniProt using EC numbers. This analysis yielded a total of 364, 198 and 463 over-expressed gene products in the cultures of *P. protegens*, *P. putida*, and the 1:1 co-culture (Table 1). The results suggest that *P. putida* leverages consortial metabolism to its advantage. The strain expresses fewer enzymes in the co-culture compared to pure cultures with the sole exception of hydrolases, which are greatly overexpressed in the co-culture. The biological significance of differential expression of hydrolases needs to be probed further. Additionally, in lieu of replication of the transcriptomics experiment, we confirmed phenotypic reproducibility by repeating the experiment to quantify biodegradation of NAFCs well over a dozen times. We observed a low standard deviation in the total amount of degradation (Figure 2) and nearly identical degradation patterns in the carbon numbers and DBEs of the NAFCs (Figure S1).

### 3.3. Assessing the Similarity between Substrates of the Expressed Enzymes and NAFCs

**Figure 4:**
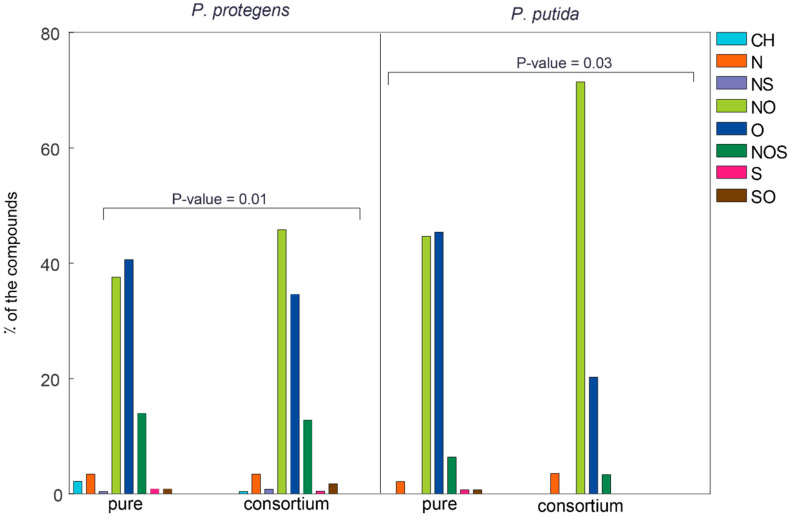
Heteroatomic classification of the substrates of the enzymes expressed by *P. protegens* and *P. putida*. NAFCs are classified as CH, N_n_, N_n_S_s_, N_n_O_o_, O_o_, N_n_O_o_S_s_, S_s_ and S_s_O_o_ based on their heteroatomic composition. These groups have been represented as CH, N, NS, NO, O, NOS, S and SO, respectively, in the legend. The metabolic networks of *P. protegens* and *P. putida* in the pure and co-cultures are significantly different. The p-values for these differences were calculated using the chi-squared test on the distribution of compounds amongst the classes. Additionally, the code used to generate this plot is published in the first Jupyter notebook that has been included in the ESI package.

We identified the canonical substrates of the enzymes expressed by the two strains in each pure culture and the 1:1 co-culture by cross-referencing their EC numbers in the MetaCyc database. We subsequently filtered the results using a number of molecular descriptors. We observed that the distribution of substrates within the different classes of the NAFCs is significantly different between each pure culture and the 1:1 co-culture (Figure 4), with *p*-values of 0.01 and 0.03 for *P. protegens* and *P. putida*, respectively.

We confirmed that O_o_ and N_n_O_o_ compounds constitute two of the largest classes of NAFCs that are degraded by the microorganisms. These compounds cumulatively account for over 75% of the compounds that are degraded by the cultures. Additionally, *P. protegens* degrades all eight classes of the NAFCs, among which CH and N_n_S_s_ compounds are uniquely degraded by the microorganism, albeit infinitesimally. Additionally, while *P. putida* degrades S_s_ and S_s_O_o_ compounds when it is cultured individually, it seems to lose this ability in the 1:1 co-culture. While we expected the metabolic networks of *P. protegens* and *P. putida* to be different, our results clearly indicate that each strain behaves differently in the pure and co-cultures.

### 3.4. Characterization of the Pathways That Metabolize NAFCs

**Figure 5:**
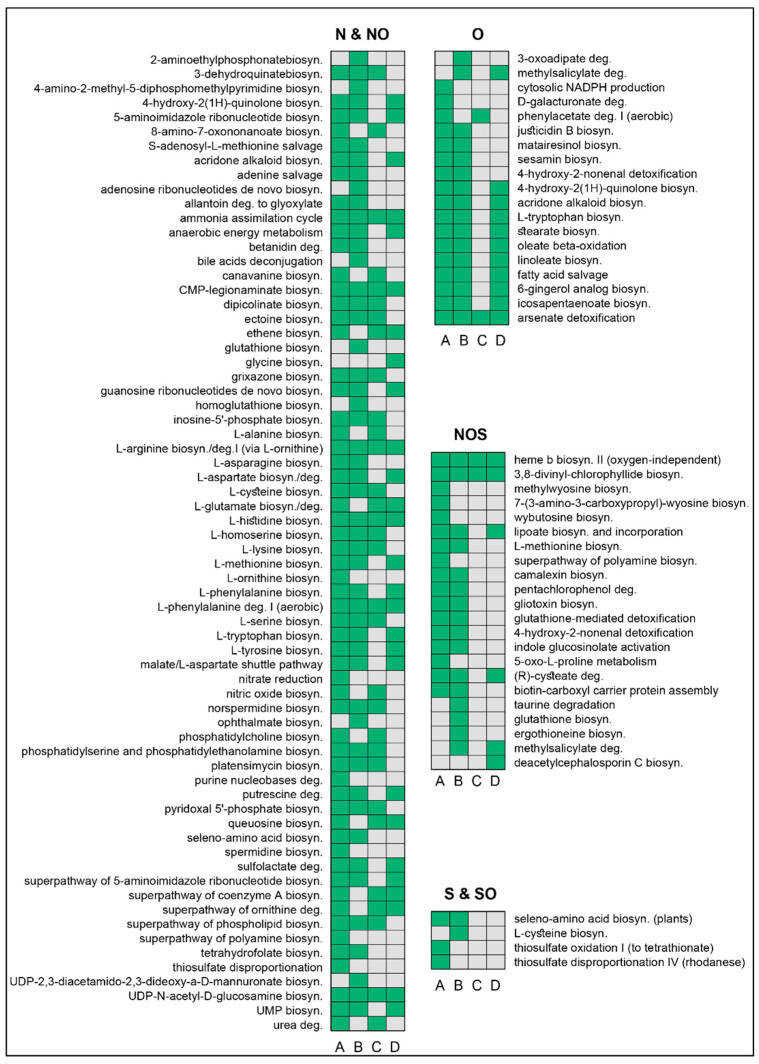
Metabolic pathways that are employed by the strains to degrade NAFCs. The compounds identified by matching the EC numbers of gene products in the RNA-seq libraries with enzymatic reactions in the MetaCyc database were categorized based on their heteroatomic composition into (1) N and NO, (2) O, (3) NOS and (4) S and SO classes. We subsequently determined the biodegradation pathways of these compounds by matching the chemical IDs (CID) of the substrates to EC numbers of putative enzymes and later interrogating all combinations of CID-EC combinations in the entire MetaCyc database. We identified pathways for (A) *P. protegens* in the 1:1 co-culture, (B) *P. protegens* cultured individually, (C) *P. putida* in the 1:1 co-culture and (D) *P. putida* cultured individually. Green boxes denote presence and grey boxes denote absence of a particular pathway. The codes used to generate this plot are published in the Jupyter notebooks that has been included in the ESI package.

We identified as many as 17 pathways in *P. protegens* that are responsible for degradation of O NAFCs when it grows in a co-culture (Figure 5). However, only two pathways—phenylacetate degradation and arsenate detoxification—were determined to be involved in degradation the same compounds by *P. putida* in the co-culture. Additionally, the 3-oxoadipate degradation pathway is only expressed by *P. protegens* in pure cultures. This pathway was initially identified in *P. putida* for its conversion of catechol and protocatechuate [52]. On the other hand, the phenylacetate degradation pathway is only expressed by both strains in the co-culture, whereas the methylsalicylate pathway is expressed by pure cultures of both strains but not in the co-culture. We hypothesize that the strains utilize the methylsalicylate pathway to convert 4-methylsalicylate and 5-methylsalicylate into 4-methylcatechol, which is then degraded via a modified ortho-cleavage pathway [53], but expression of the pathway in only the pure cultures is an intriguing observation. Similarly, the cytosolic NADPH production and D-galacturonate degradation pathways are only detected in *P. protegens* in the 1:1 co-culture.

The metabolism of N and NO compounds could involve the participation of as many as 59 pathways in *P. protegens* in the 1:1 co-culture and as few as 27 pathways in pure cultures of *P. putida*. Six pathways—L-ornithine biosynthesis, nitrate reduction, purine nucleobases degradation, spermidine biosynthesis, the polyamine biosynthetic superpathway and thiosulfate disproportionation—are uniquely expressed by *P. protegens* in the co-culture. On the other hand, pure cultures of the same host express six unique pathways for biosynthesis of 2-aminoethylphosphonate, adenosine ribonucleotides, UDP-2,3-diacetamido-2,3-dideoxy-α-D-mannuronate, glutathione, homoglutathione, ophthalmate and 4-amino-2-methyl-5-diphosphomethylpyrimidine, as well as a unique pathway for deconjugation of bile acids. The only pathway that is uniquely detected in pure cultures of *P. putida* is the glycine biosynthetic pathway, a single reaction pathway comprising the metabolite 5,10-methylenetetrahydropteroyl mono-L-glutamate. The NOS pathways for oxygen-independent biosynthesis of heme b and the biosynthesis of 3,8-divinyl-chlorophyllide were detected in all four samples. Additionally, *P. protegens* cells in the consortium express five unique pathways for degradation of NOS compounds, including the methylwyosine, 7-(3-amino-3-carboxypropyl)-wyosine and wybutosine biosynthetic pathways, the superpathway for polyamine biosynthesis and a pathway for 5-oxo-L-proline metabolism.

Pure cultures of *P. protegens* also uniquely express the taurine degradation pathway and the biosynthetic pathways for ergothioneine and glutathione, whereas pure cultures of *P. putida* uniquely express the biosynthetic pathway for deacetylcephalosporin C. Herein, isopenicillin N is isomerized to penicillin N, following which the penam thiazolidine ring is oxidatively opened and expanded to yield a six-membered dihydrothiazine ring. Incidentally, the pure cultures of both strains express methylsalicylate degradation pathway. Lastly, the only unique pathways that play a role in metabolism of S and SO compounds were detected in cultures of *P. protegens*. Of these, three pathways were uniquely expressed by *P. protegens* cells in the co-culture, whereas the L-cysteine biosynthetic pathway is uniquely detected in the pure culture.

### 3.5. Analysis of Differential Expression in Pure and Co-Cultures of the Pseudomonads

**Figure 6:**
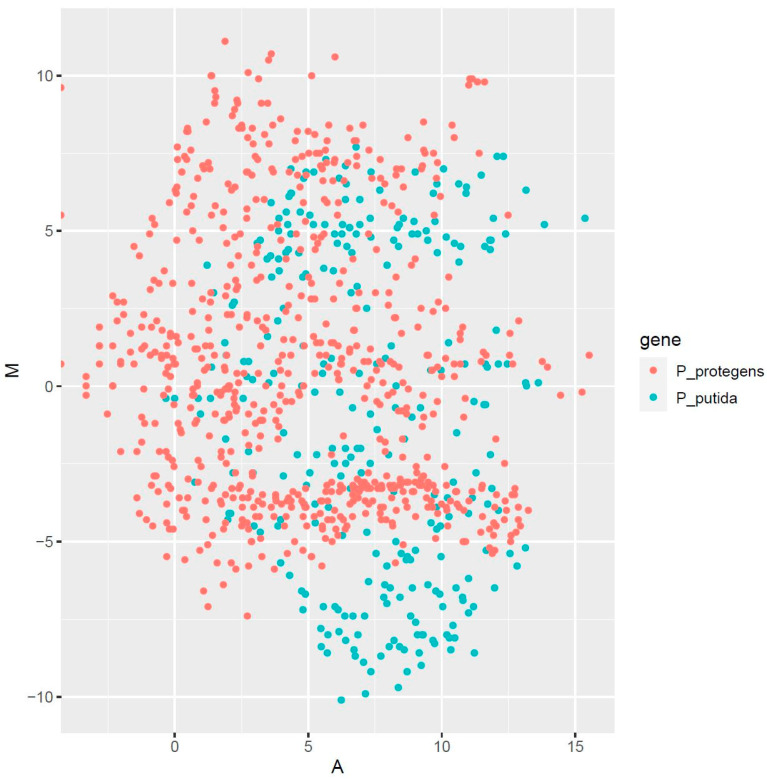
MA plot for differential expression of the genes. M is estimated as log_2_(TPM of the consortium/TPM of the individual strains), whereas A is determined as 0.5 × log_2_(TPM of the consortium x TPM of the individual strains). These values are calculated for all up-regulated genes.

We estimated the ratio between the TPM values of each pseudomonad in the 1:1 co-culture and each pure culture. The use of suppression subtractive hybridization (SSH) for generating the RNA-seq libraries ensures that the calculated TPM values for the tester samples adequately measure the up-regulated genes. Genes with a positive log2 fold-change (*viz.* increased TPM values in the co-culture) are enriched and probably play a greater role in metabolism of NAFC in the co-culture compared to the pure cultures, which provides useful insights on synergistic metabolism by the strains. Similarly, the MA plot for differential expression of the genes reveals that the distribution of expression is more or less symmetrical (Figure 6). We also cross-referenced the genes exhibiting the greatest changes in TPM between the 1:1 co-culture and the pure cultures of both strains in the database of KEGG pathways to shed light on how the metabolic networks of both strains adapt during co-culturing (the top five hits are listed in Table 2 and Table 3, all pathways have been summarized in Table S4 in the ESI). Additionally, we have also compiled the maps of the top five pathways in Figure S2, which has been uploaded to the article’s webpage as a separate file.

## 4. Discussion

We investigated the transcriptomic responses of pure and 1:1 co-culture of two pseudomonads, *P. protegens* and *P. putida* after exposure to a concentrated mixture of NAFCs extracted from OSPW. Both strains were isolated from OSPW.

Unlike previous studies on microbial degradation of OSPW that solely focused on either quantifying the composition of the microbial consortium before and after degradation of NAFCs or confirming specific metabolic activities in the native microbiome of OSPW or deciphering the mechanisms of biodegradation of individual model NAFCs by varying their structures [37,54,55], our investigation is the first of its kind to co-analyze transcriptomic, metabolite and toxicity data and elucidate the metabolic networks involved in degradation of all heteroatom classes of NAFCs by pure and co-cultures of the two strains. We established an entirely novel bioinformatics workflow to achieve this objective and deduced that the molecular distribution of the substrates that are degraded by both pure cultures and the 1:1 co-culture are significantly different.

We also confirmed that the 1:1 co-culture degrades a higher quantity of NAFCs compared to pure cultures of the two pseudomonads. The co-culture also completely detoxifies OSPW after 30 days of treatment. Additionally, *P. protegens* showed an over-representation of fatty acids, valine, leucine and isoleucine degradation pathways, which can be relevant to the degradation of linear NAs and nitrogen containing NAFCs, respectively. Our analysis also revealed that GO terms such as carbohydrate metabolism, oxidoreductase activity, purine biosynthesis and metabolic pathways involved in processing macromolecules, heterocyclic compounds and organic cyclic compounds are significantly over-represented in the samples. The data also indicate that the strains employ a variety of oxidoreductases, hydrolases and ligases to metabolize the NAFCs. Finally, our experimental observation that the 1:1 co-culture is stable is corroborated by the KEGG pathway analysis, which reveals how the metabolic network for NAFCs is shared between the two strains. Our findings indicate that the potential co-operative interaction between isolated *P. protegens* and *P. putida* can be further extended to biodegradation of more NAFCs.

In closing, our work represents a significant advance for the field of microbial bioremediation that opens previously unavailable opportunities to rationally engineer or adaptively evolve more strains with improved phenotypic traits. In particular—and courtesy of the mainstreaming of metagenomic screening—one could replace rate-limiting enzymes in the identified degradation pathways with heterologues that exhibit higher k_cat_/K_m_ values. Enzyme engineering too can be employed to alter an enzyme’s substrate specificity, cofactor dependency, enhance k_cat_ and/or lower K_m_. In light of the poor solubility of NAs in water, we believe that achieving lower K_m_ values is arguably more important than achieving higher k_cat_ values since microbial growth in tailings ponds will also be constrained by the availability of iron, phosphate and nitrogen, among other variables. To that end, one could employ adaptive laboratory evolution (ALE) to selectively improve the phenotype of environmental strains by culturing them in concentrated mixtures of NAs in a chemostat or batch reactor for prolonged durations [56]. ALE traces its roots to the pioneering work by Senior et al., who successfully employed the method to evolve *P. putida* to express a more active dehalogenase, thereby permitting it to grow on the herbicide Dalapon [57].

## Figures and Tables

**Table 1:** Summary of the number of genes (categorized based on enzyme class) that are expressed by the three cultures in response to exposure to NAFCs.

Enzyme Class	*P. putida*	*P. putida* in Co-Culture	*P. protegens*	*P. protegens* in Co-Culture
EC 1—Oxidoreductases	41	25	72	62
EC 2—Transferases	54	40	122	126
EC 3—Hydrolases	70	77	276	305
EC 4—Lyases	15	6	23	25
EC 5—Isomerases	15	6	18	19
EC 6—Ligases	21	10	33	19
EC 7—Translocases	16	6	23	17

**Table 2:** KEGG hits for pathways expressed by *P. protegens* in the 1:1 co-culture that exhibit the greatest changes in TPM compared to its metabolic activity in pure cultures.

**Top 5 Pathways with the Greatest Increase in TPM**			
**Name**	**ID**	**Enzyme Count**	**Genes**
Fatty acid degradation	map00071	8	fadB_2, betB_2, fadD_3, mmgC_1, dmdC_5
Valine, leucine and isoleucine degradation	map00280	7	davT_1, fadB_2, betB_2, dmdC_5, aidB, lpdG_2
Glycolysis/gluconeogenesis	map00010	6	betB_2, aceE, ppsA, cbbA, lpdG_2
Tryptophan metabolism	map00380	6	fadB_2, betB_2, mmgC_1, DJCOHBMJ_04543, lpdG_2
Amino sugar and nucleotide sugar metabolism	map00520	6	wbpA, capD, rkpK_1, gtaB, glmM, gtaB
**Top 5 Pathways with the Greatest Decrease in TPM**			
**Name**	**ID**	**Enzyme Count**	**Genes**
Purine metabolism	map00230	10	recA, uvrD, zapE_3, lepA, uvrA_2, rep_2, lon_1, DJCOHBMJ_04023, apxIB, ettA, gyrB, ffh, DJCOHBMJ_04017, recD, amn, cysC_1guaA, relA_2, apt, DJCOHBMJ_06051, nrdB, purL, hpt
Propanoate metabolism	map00640	10	fadJ_2, acnD, menB_1, bauC_1, accD, bkdA2, acs, bauC_1, lpdG_1, sucD
Carbon fixation pathways in prokaryotes	map00720	7	fadJ_2, accD, acs, acnD, fumC_1, sucD
Valine, leucine and isoleucine degradation	map00280	6	fadJ_2, menB_1, bauC_1, bkdA2, lpdG_1
Drug metabolism—other enzymes	map00983	6	yfcG_2, gstB_1, guaA, rnk_1, ileS, nrdB, hpt

**Table 3:** KEGG hits for pathways expressed by *P. putida* in the 1:1 co-culture that exhibit the greatest changes in TPM compared to its metabolic activity in pure cultures.

**Top 5 Pathways with the Greatest Increase in TPM**			
**Name**	**ID**	**Enzyme Count**	**Genes**
Pyruvate metabolism	map00620	5	maeB
Glutathione metabolism	map00480	3	ldc, ggt_1, ggt_1
Aminoacyl-tRNA biosynthesis	map00970	3	metG, lysS, ndk
Arginine and proline metabolism	map00330	3	ldc, map_1, HNFJGDPB_03903
Lysine biosynthesis	map00300	2	dapL
**Top 5 Pathways with the Greatest Decrease in TPM**			
**Name**	**ID**	**Enzyme Count**	**Genes**
Glycolysis/Gluconeogenesis	map00010	4	pckA, pfkB
Pyruvate metabolism	map00620	3	pckA, hchA
Arginine biosynthesis	map00220	3	gdhB, glnA
Amino sugar and nucleotide sugar metabolism	map00520	3	wbpI, pfkB
Nitrogen metabolism	map00910	3	gdhB, glnA

## Data Availability

All raw short and long reads and RNA-seq reads have been deposited to the NCBI Sequence Read Archive (SRA). The deposited data can be accessed through the NCBI BioProject PRJNA669150.

## Supplementary Materials

The following are available online at https://www.mdpi.com/article/10.3390/microorganisms9102124/s1. The accompanying ESI package includes (1) characterization of the NAFCs using HPLC-Orbitrap; (2) genome sequencing and assembly; (3) heatmaps of NAFCs after degradation by pure cultures of *P. putida*, *P. protegens* and the 1:1 co-culture (Figure S1); (4) genome assembly statistics for *P. protegens* and *P. putida* (Table S1); (5) summary of sequencing of the RNA-seq libraries (Table S2); (6) all enriched GO terms for the upregulated genes in *P. protegens*, *P. putida*, and the 1:1 co-culture (Table S3); (7) KEGG pathway mapping results of the genes showing an increase and decrease in TPM counts for *P. protegens* and *P. putida* in the 1:1 co-culture compared to their pure cultures (Table S4); (8) KEGG pathway maps for the results summarized in Table S4 (Figure S2); and (9) Python codes, input and output files to generate the substrates of the enzymes related to NAFCs and characterize the biodegradation pathways as an ancillary zip file. Figure S2 has been uploaded as a separate file.
