# Supplementary material for: Transcriptome Analysis of Environmental Pseudomonas Isolates Reveals Mechanisms of Biodegradation of Naphthenic Acid Fraction Compounds (NAFCs) in Oil Sands Tailings
Source: Microorganisms. 2021 Oct 9;9(10):2124. doi: 10.3390/microorganisms9102124 (PMC8540809; doi:10.3390/microorganisms9102124)
Supplement: Supplementary file 1 [file microorganisms-09-02124-s001.zip › supplementary/Microorganisms - August 2021 - ESI for manuscript.pdf]

Electronic Supporting Information

Transcriptome Analysis of Environmental *Pseudomonas* Isolates Reveals Mechanisms of Biodegradation of Naphthenic Acid Fraction Compounds (NAFCs) in Oil Sands Tailings

Parisa Chegounian[1], Stephane Flibotte[2], Kerry M. Peru[3],

John V. Headley[3], Dena W. McMartin[4], Bryne Gramlich[5] and Vikramaditya. G. Yadav[1,6,*]

[1]Department of Chemical and Biological Engineering, The University of British Columbia, Vancouver, BC, Canada

[2]UBC/LSI Bioinformatics Facility, University of British Columbia, Vancouver, BC Canada

[3]Watershed Hydrology and Ecology Research Division, Water & Science Technology Directorate, Environment Canada, Saskatoon, SK, Canada

[4]Department of Civil, Geological & Environmental Engineering, University of Saskatchewan, Saskatoon, SK, Canada

[5]Allonnia, Boston, MA, USA

[6]School of Biomedical Engineering, The University of British Columbia, Vancouver, BC, Canada

*Corresponding author: vikramaditya.yadav@ubc.ca

Contents

Materials and methods **3**

Characterization of NAFCs using HPLC-Orbitrap mass spectrometry 3

Genome sequencing and assembly 4

Figures **5**

Figure S1: The heatmaps of NAs 5

Tables **5**

Table S1: The genome assembly statistics 5

Table S2: Summary of sequencing of RNA-Seq libraries 6

References **6**

Materials and methods

Characterization of NAFCs using HPLC-Orbitrap mass spectrometry. Component separation was performed on a Thermo Fisher Ultimate 3000 HPLC system using a C8 column (150 x 3.0 mm, 3 μm particle size) and the eluent was directly injected into an Orbitrap Elite mass spectrometer. Component separation was performed on a Thermo Fisher Ultimate 3000 HPLC system using a C8 column (150 x 3.0 mm, 3 μm particle size) and the eluent was directly injected into an Orbitrap Elite mass spectrometer. The HPLC column was maintained at 40°C and flow rate of the mobile phase was set at 0.5 mL/min. The sample injection volume was 5 μL. The mobile phase was a gradient of two solvents, a 0.1% acetic acid in 90%-10% (by volume) solution of water and methanol, respectively, (solvent A); and methanol (solvent B). The solvent gradient was 5% B for 1 minute, followed by a linear ramp to 90% B over the next 9 minutes, a ramp to 99% B over the next 5 minutes, a return to 5% B in the next minute, and a final hold at this condition for 4 minutes. The total HPLC run time was 20 minutes. The Orbitrap was operated at a source temperature of 350°C in negative electrospray ionization (ESI^-) mode with sheath, auxiliary and sweep gas flow at 30, 5 and 5 (arbitrary units), respectively. The capillary temperature and S-Lens RF were held at 350°C and 65 %, respectively, and the resolving power was set to a nominal value of 120,000 at a full width half-maximum at m/z 400 and using a full maximum ion time of 200 milliseconds. All chromatograms were processed in Xcalibur 2.2 QuanBrowser using the same data processing parameters. Manual integration was performed only when necessary. A technical mixture of NAs that is marketed by the Merichem Company was used to generate calibration curves, and authentic, pure, isotopically labelled dodecanoic-D23 acid was used as the internal calibration standard. The internal standard was injected into all blanks, standards and samples. We estimated the peak area ratios for all NAs with C5-C22 and DBEs ranging between 1 and 10. The peak area ratio of an analyte is calculated by dividing the

peak area of the species with the peak area of the internal standard, and the peak area ratios were then fitted to a least-squares regression model. The total peak area ratio for all NAs was then interpolated using a linear calibration function, which ultimately yielded the total concentration of all NAs in the sample.

Genome sequencing and assembly. Target coverage was100X depth for both short and long read libraries. Short and long reads were quality controlled with fastQC (https://www.bioinformatics.babraham.ac.uk/projects/fastqc) and Nanostat (De Coster et al., 2018), respectively. Illumina reads were down sampled to 100X depth using bbnorm (http://sourceforge.net/projects/bbmap). Nanopore reads were adapter trimmed with Porechop (https://github.com/rrwick/Porechop), then filtered to a minimum length 1500 and down sampled to an expected genome coverage of 50X using Filtlong (https://github.com/rrwick/Filtlong). We used the canu assembler (Sergey Koren, Brian P. Walenz, Konstantin Berlin, Jason R. Miller, Nicholas H. Bergman, 2017) with error correction with Pilon (Walker et al., 2014) as well as the Unicycler assembler (Wick et al., 2017) in hybrid mode for prokaryotic genomes. Both genomes were polished using ntEdit (Warren et al., 2019)as well as a short-read correction approach. We quality controlled genomes using Quast (Gurevich et al., 2013) for overall statistics (size, GC content, contiguity) as well as BUSCO (Simão et al., 2015) for essential gene completeness scores. Both assemblies were compared to pre-existing reference genomes using Quast and the best one was selected based on a combination of contiguity, size, completeness, and misassembly rates (**Table S1**). Incidentally, we detected a significant level of human mitochondrial genetic contamination in the *P. putida* RNA-seq library, which resulted in impaired detection of upregulated bacterial genes in the tester samples. As a consequence, we initially aligned the reads to the human reference genome hg38 using HISAT2 (Kim et al., 2019) and subsequently extracted the unmapped read pairs using the SAMtools toolbox (Li et al., 2009). We believe that the contamination accounts for some of the differences in the numbers of upregulated

genes in pure cultures of the *Pseudomonas* isolates. On the other hand, the RNA-seq library of the 1:1 co-culture was free of contamination.

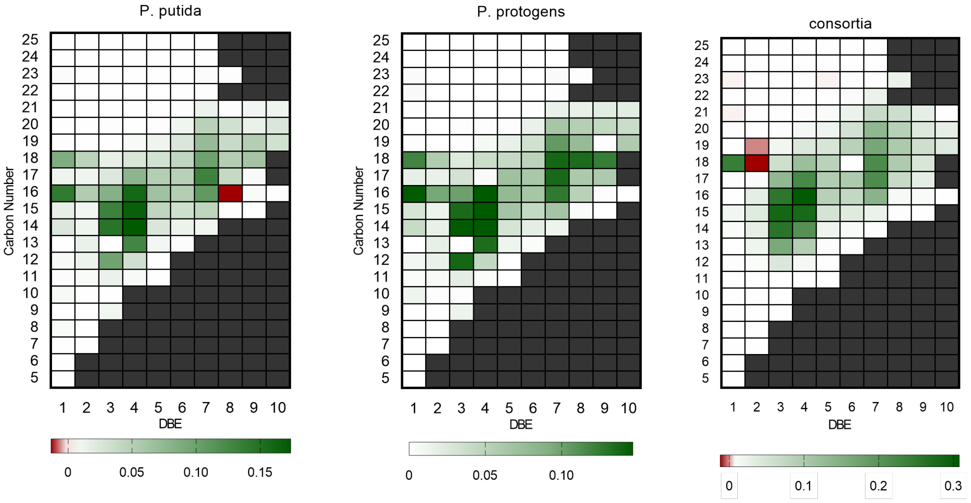

Figure S1. The heatmaps of NAs with general formula of $C_cH_hN_nO_oS_s$; Degraded concentration (mg/L) of identified NAi (CcH2c+ZO2 and total concentration of 28.2 mg/L, $DBE = c - \frac{2c+Z}{2} + 1$) compounds is measured as ΔCi degraded by pure and consortial cultures; the white cells represent the available compounds with concentrations below the detection limit of LC-Orbitrap and the black boxes shows the absence of that DBE-carbon number combinations in the samples; red boxes indicate the compounds with increase in the concentration after biodegradation.

Table S1. The genome assembly statistics for *P. protegens* and *P. putida*.

Genomic DNA	# contigs (>= 10000 bp)	Comments	GC (%)	method	% complete	missing %
P. protegens	1	A single 6.7 MB chromosome	62.13	unicycler	100	0
P. putida	1	A single 5.7 MB chromosome	61.91	canu	100	0

Table S2. Summary of sequencing of RNA-Seq libraries for pure *P. protegens* and *P. putida,* and their consortial cultures in exposure to NAFCs

RNA-Seq library	PF Clusters	Yield (Mbases)	%>=Q30	Mean Quality
P. protegens	114,427,134	34,557	82.44	35.7
P. putida	128,138,156	38,698	80.11	35.07
Consortial culture	130,083,429	39,285	78.59	34.68

References

De Coster, W., D'Hert, S., Schultz, D.T., Cruts, M., Van Broeckhoven, C., 2018. NanoPack: Visualizing and processing long-read sequencing data. Bioinformatics 34, 2666–2669. https://doi.org/10.1093/bioinformatics/bty149

Gurevich, A., Saveliev, V., Vyahhi, N., Tesler, G., 2013. QUAST: Quality assessment tool for genome assemblies. Bioinformatics 29, 1072–1075. https://doi.org/10.1093/bioinformatics/btt086

Sergey Koren, Brian P. Walenz, Konstantin Berlin, Jason R. Miller, Nicholas H. Bergman, and A.M.P., 2017. Canu: scalable and accurate long-read assembly via adaptive k-mer weighting and repeat separation. Genome Research 27, 722–736. https://doi.org/10.1101/gr.215087.116.Freely

Simão, F.A., Waterhouse, R.M., Ioannidis, P., Kriventseva, E. V., Zdobnov, E.M., 2015. BUSCO: Assessing genome assembly and annotation completeness with single-copy orthologs. Bioinformatics 31, 3210–3212. https://doi.org/10.1093/bioinformatics/btv351

Walker, B.J., Abeel, T., Shea, T., Priest, M., Abouelliel, A., Sakthikumar, S., Cuomo, C.A., Zeng, Q., Wortman, J., Young, S.K., Earl, A.M., 2014. Pilon: An integrated tool for comprehensive microbial variant detection and genome assembly improvement. PLoS ONE 9. https://doi.org/10.1371/journal.pone.0112963

Warren, R.L., Coombe, L., Mohamadi, H., Zhang, J., Jaquish, B., Isabel, N., Jones, S.J.M., Bousquet, J., Bohlmann, J., Birol, I., 2019. ntEdit: scalable genome sequence polishing. Bioinformatics (Oxford, England) 35, 4430–4432.

Wick, R.R., Judd, L.M., Gorrie, C.L., Holt, K.E., 2017. Unicycler: Resolving bacterial genome assemblies from short and long sequencing reads. PLoS Computational Biology 13, 1–22.
