# Supplementary figures and images for: Transcriptome Analysis of Environmental Pseudomonas Isolates Reveals Mechanisms of Biodegradation of Naphthenic Acid Fraction Compounds (NAFCs) in Oil Sands Tailings
Source: Microorganisms. 2021 Oct 9;9(10):2124. doi: 10.3390/microorganisms9102124 (PMC8540809; doi:10.3390/microorganisms9102124)

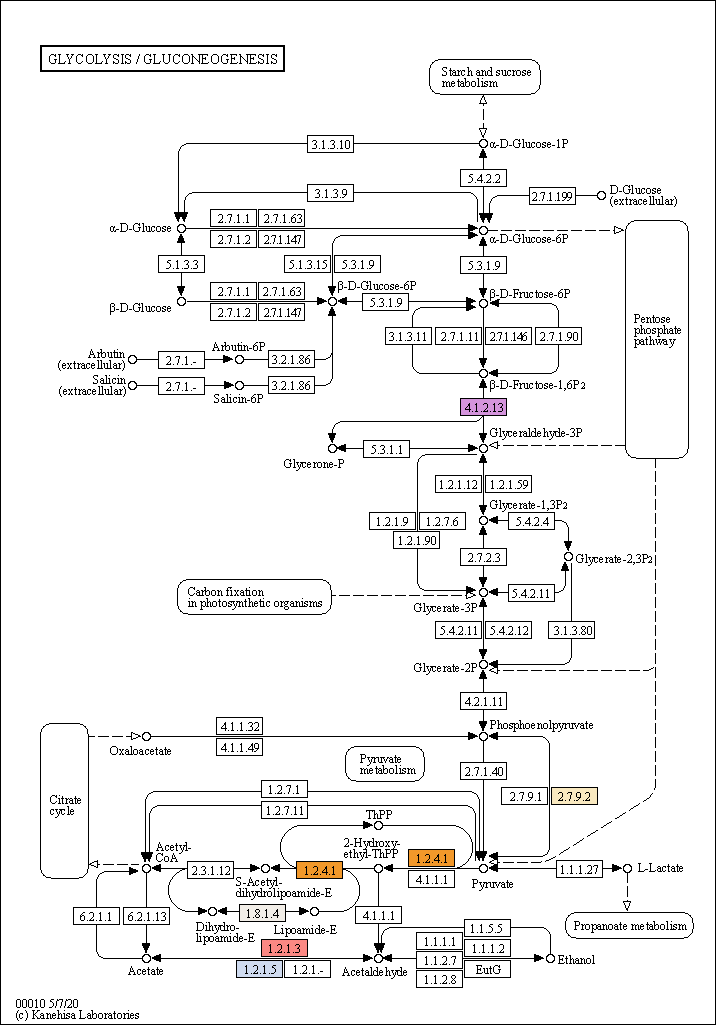

Supplement: Supplementary file 1 [file microorganisms-09-02124-s001.zip › supplementary/Figure S2/Figure S2/KEGG_Pathways maps_protegens consortia vs. pure/high_kegg_map00010_glycolysis_gluconeogenesis_20200910_143146.png]

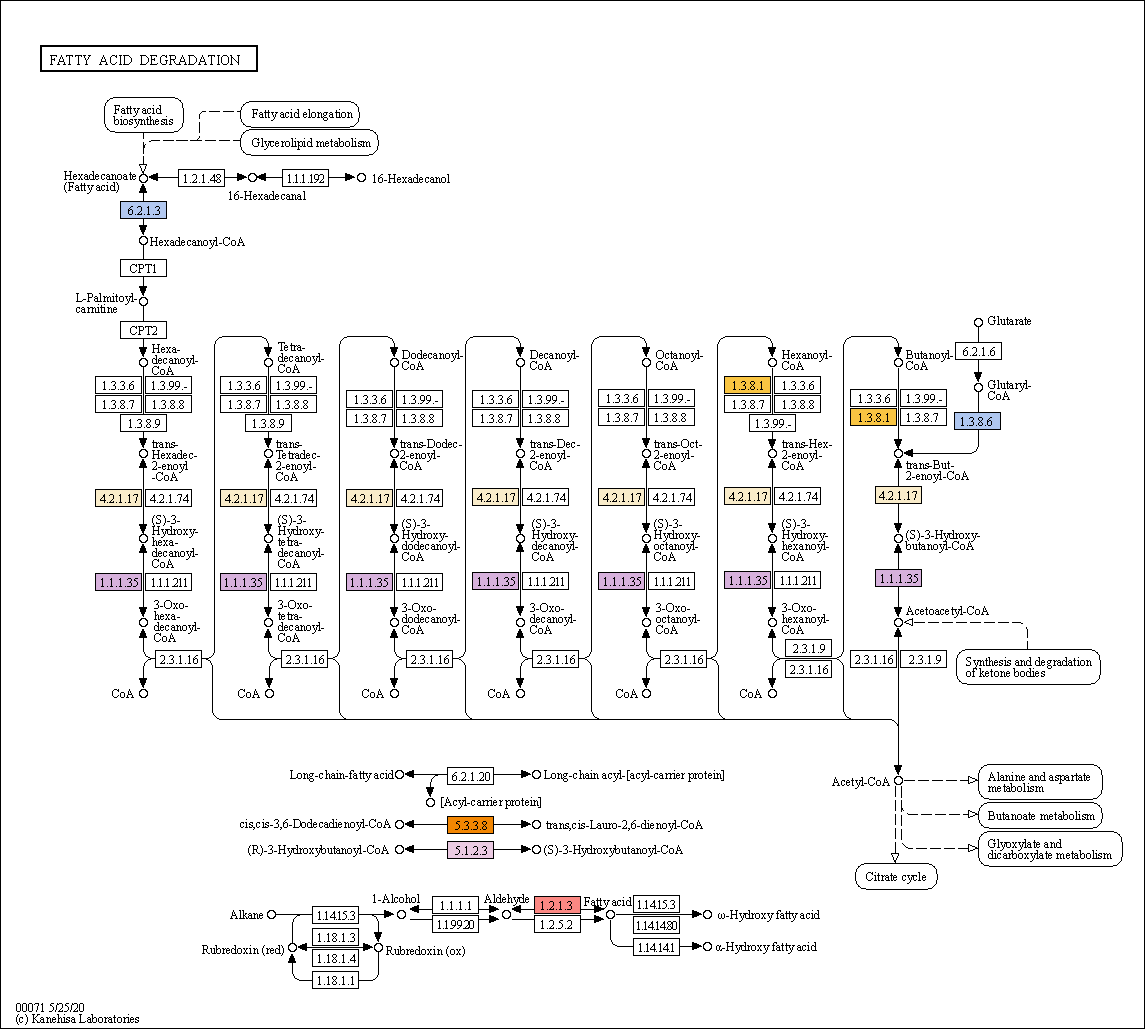

Supplement: Supplementary file 1 [file microorganisms-09-02124-s001.zip › supplementary/Figure S2/Figure S2/KEGG_Pathways maps_protegens consortia vs. pure/high_kegg_map00071_fatty_acid_degradation_20200910_143158.png]

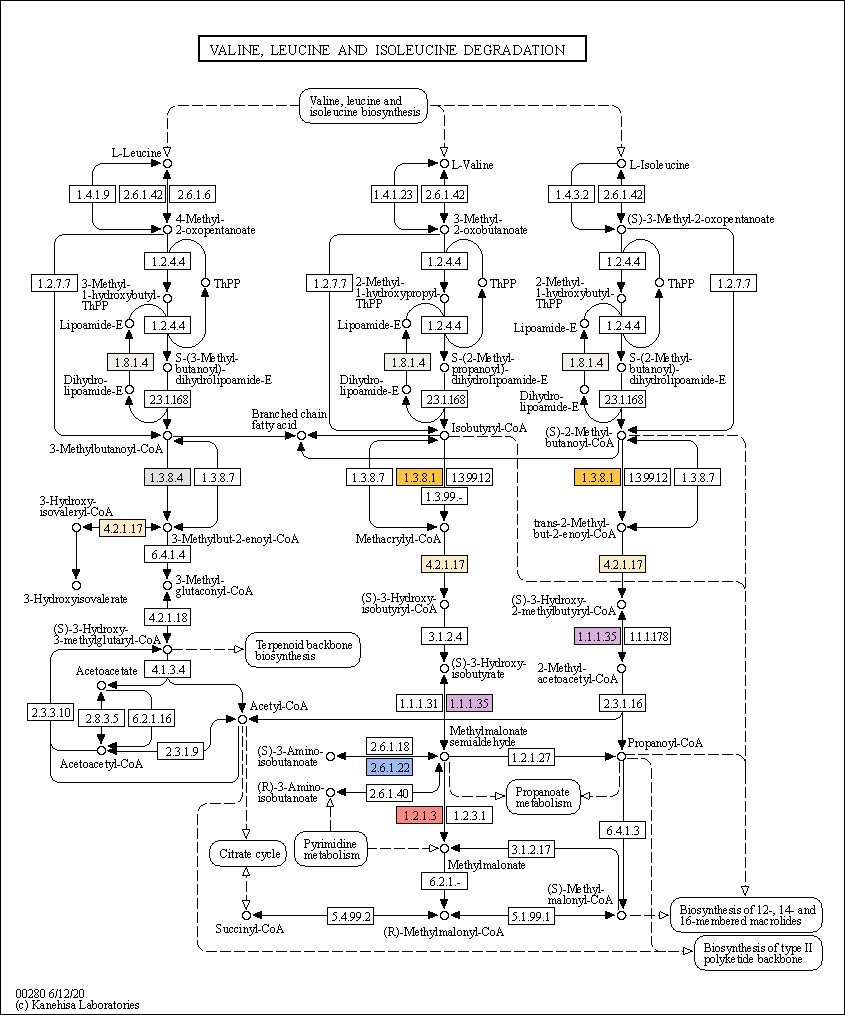

Supplement: Supplementary file 1 [file microorganisms-09-02124-s001.zip › supplementary/Figure S2/Figure S2/KEGG_Pathways maps_protegens consortia vs. pure/high_kegg_map00280_valine_leucine_and_isoleucine_degradation_20200910_143150.png]

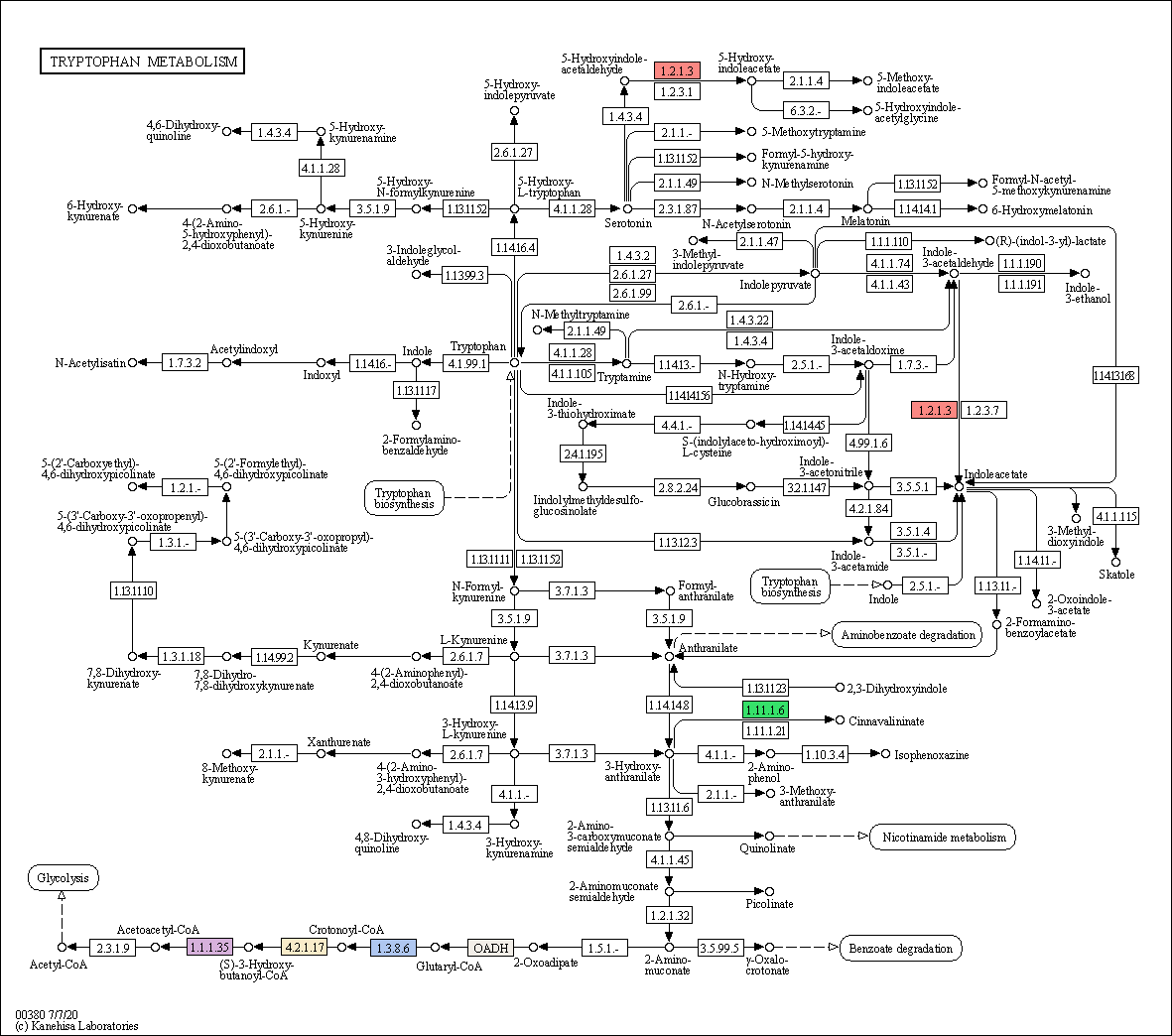

Supplement: Supplementary file 1 [file microorganisms-09-02124-s001.zip › supplementary/Figure S2/Figure S2/KEGG_Pathways maps_protegens consortia vs. pure/high_kegg_map00380_tryptophan_metabolism_20200910_143146.png]

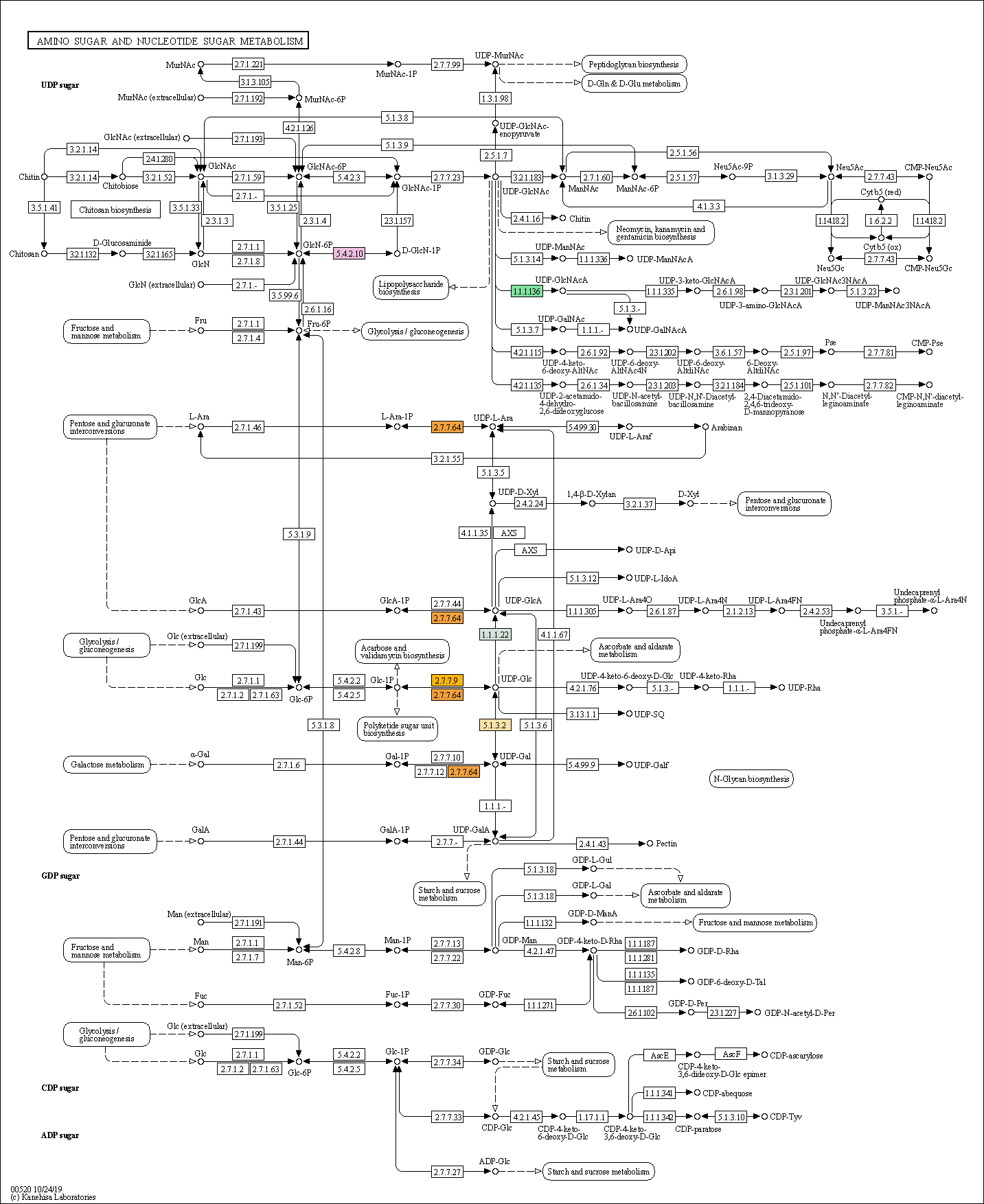

Supplement: Supplementary file 1 [file microorganisms-09-02124-s001.zip › supplementary/Figure S2/Figure S2/KEGG_Pathways maps_protegens consortia vs. pure/high_kegg_map00520_amino_sugar_and_nucleotide_sugar_metabolism_20200910_143151.png]

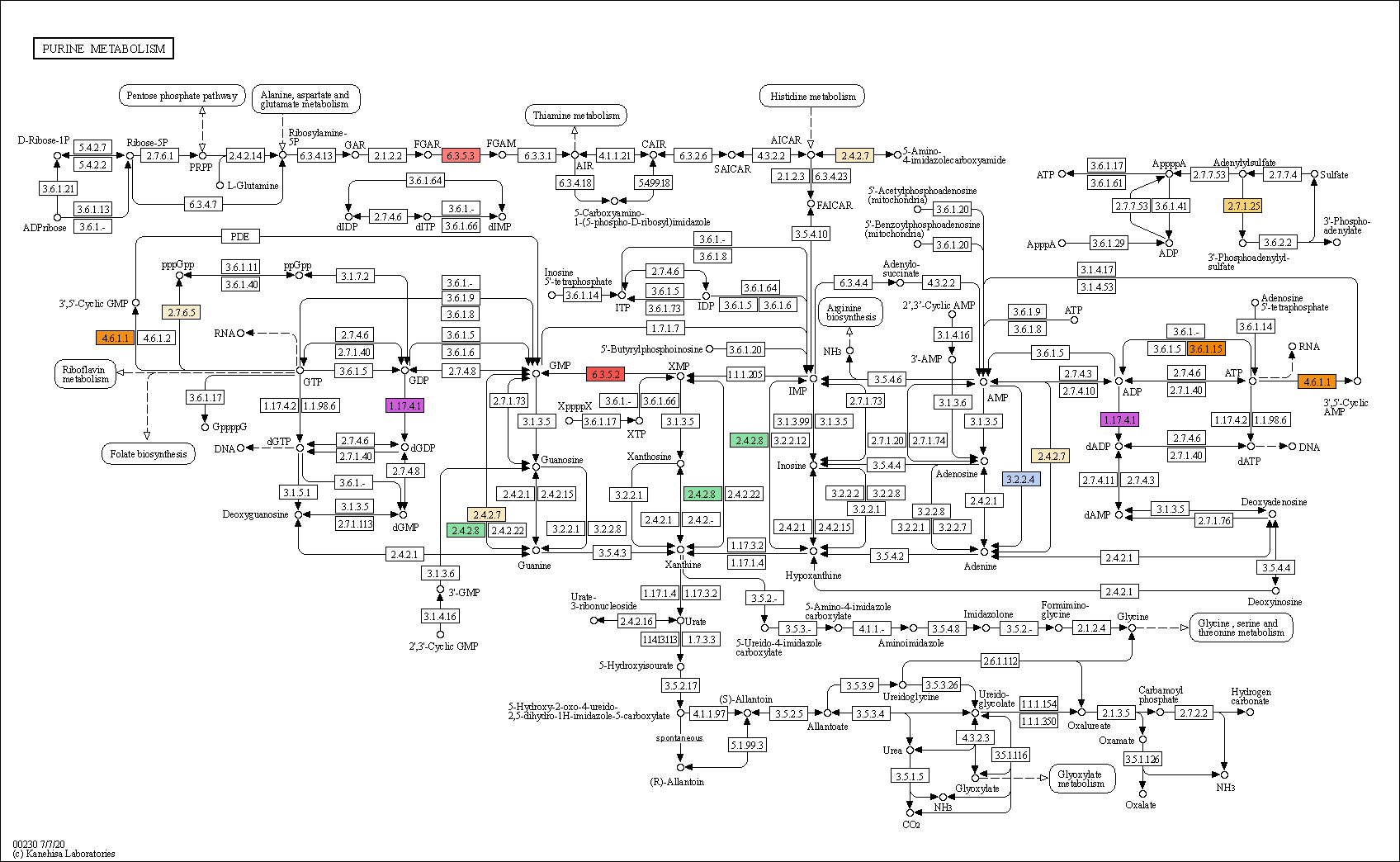

Supplement: Supplementary file 1 [file microorganisms-09-02124-s001.zip › supplementary/Figure S2/Figure S2/KEGG_Pathways maps_protegens consortia vs. pure/low_kegg_map00230_purine_metabolism_20200910_143121.png]

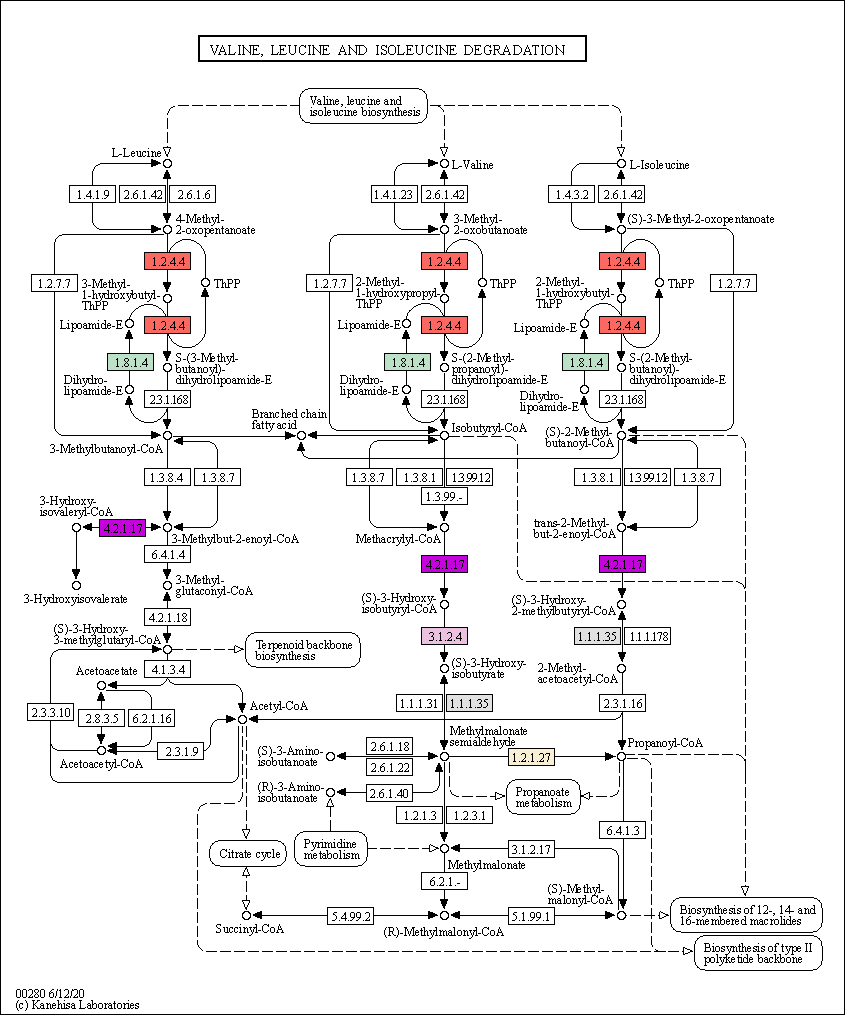

Supplement: Supplementary file 1 [file microorganisms-09-02124-s001.zip › supplementary/Figure S2/Figure S2/KEGG_Pathways maps_protegens consortia vs. pure/low_kegg_map00280_valine_leucine_and_isoleucine_degradation_20200910_143101.png]

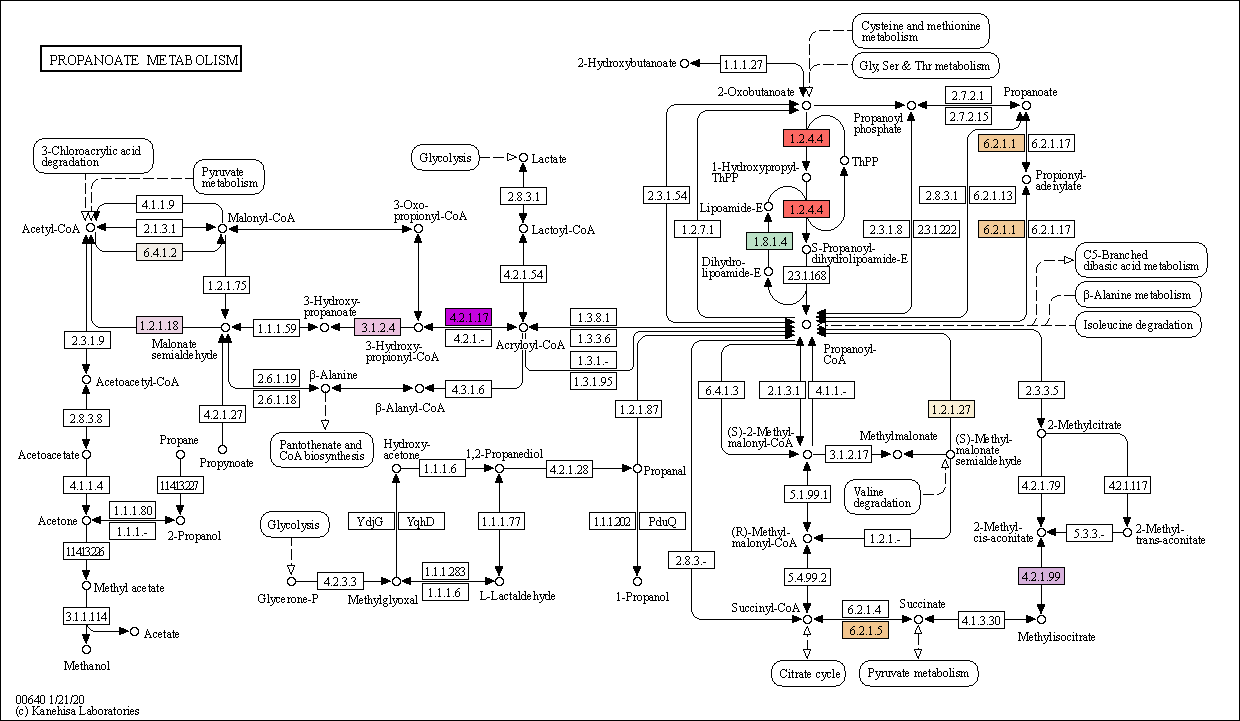

Supplement: Supplementary file 1 [file microorganisms-09-02124-s001.zip › supplementary/Figure S2/Figure S2/KEGG_Pathways maps_protegens consortia vs. pure/low_kegg_map00640_propanoate_metabolism_20200910_143128.png]

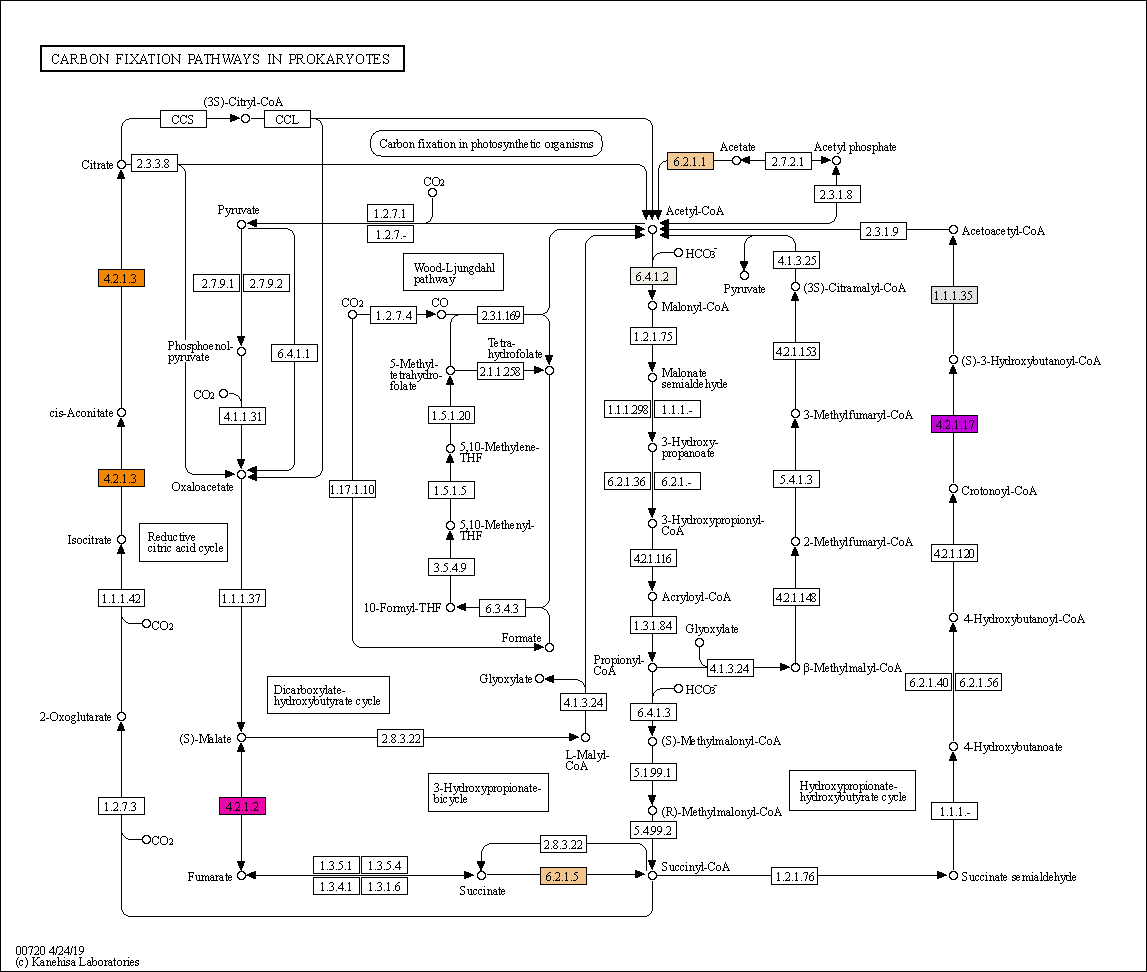

Supplement: Supplementary file 1 [file microorganisms-09-02124-s001.zip › supplementary/Figure S2/Figure S2/KEGG_Pathways maps_protegens consortia vs. pure/low_kegg_map00720_carbon_fixation_pathways_in_prokaryotes_20200910_143126.png]

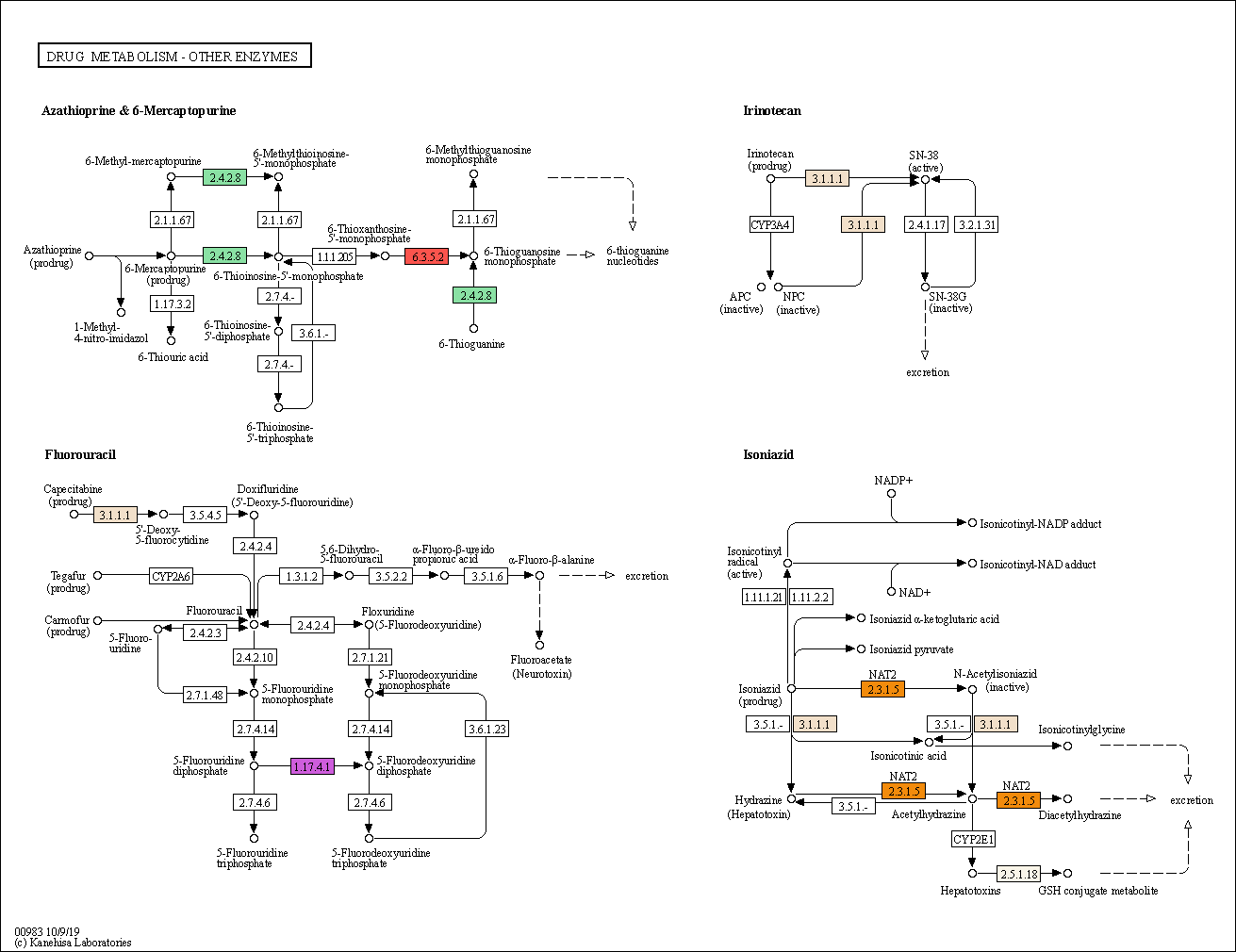

Supplement: Supplementary file 1 [file microorganisms-09-02124-s001.zip › supplementary/Figure S2/Figure S2/KEGG_Pathways maps_protegens consortia vs. pure/low_kegg_map00983_drug_metabolism_other_enzymes_20200910_143112.png]

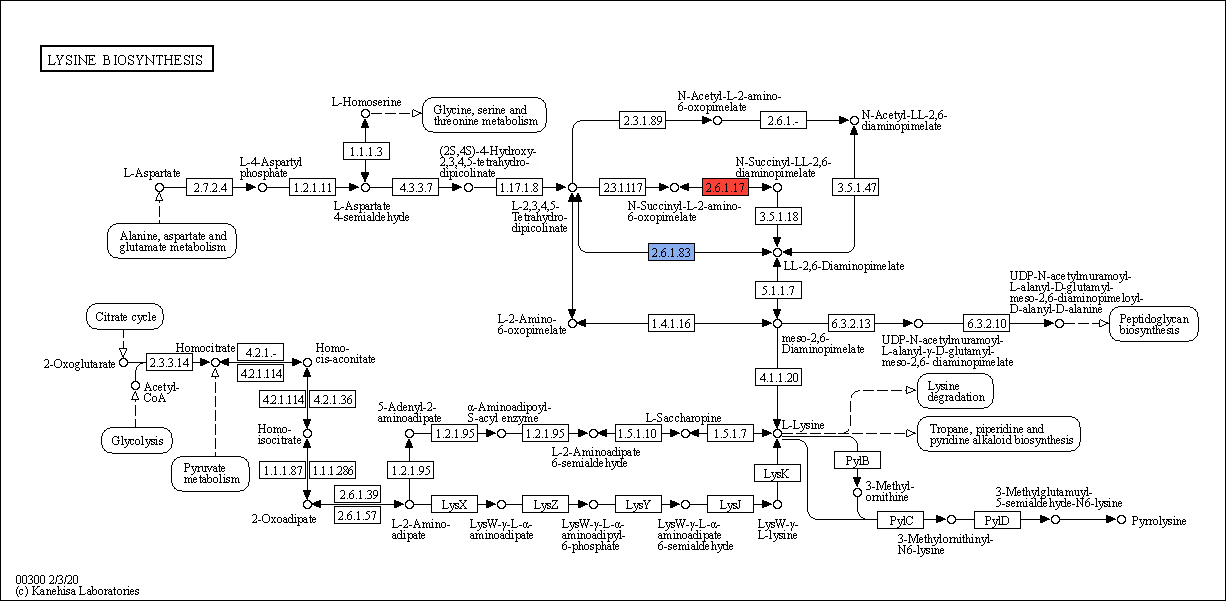

Supplement: Supplementary file 1 [file microorganisms-09-02124-s001.zip › supplementary/Figure S2/Figure S2/KEGG_Patwhays maps_putida consortia vs. pure/high_kegg_map00300_lysine_biosynthesis_20200910_142911.png]

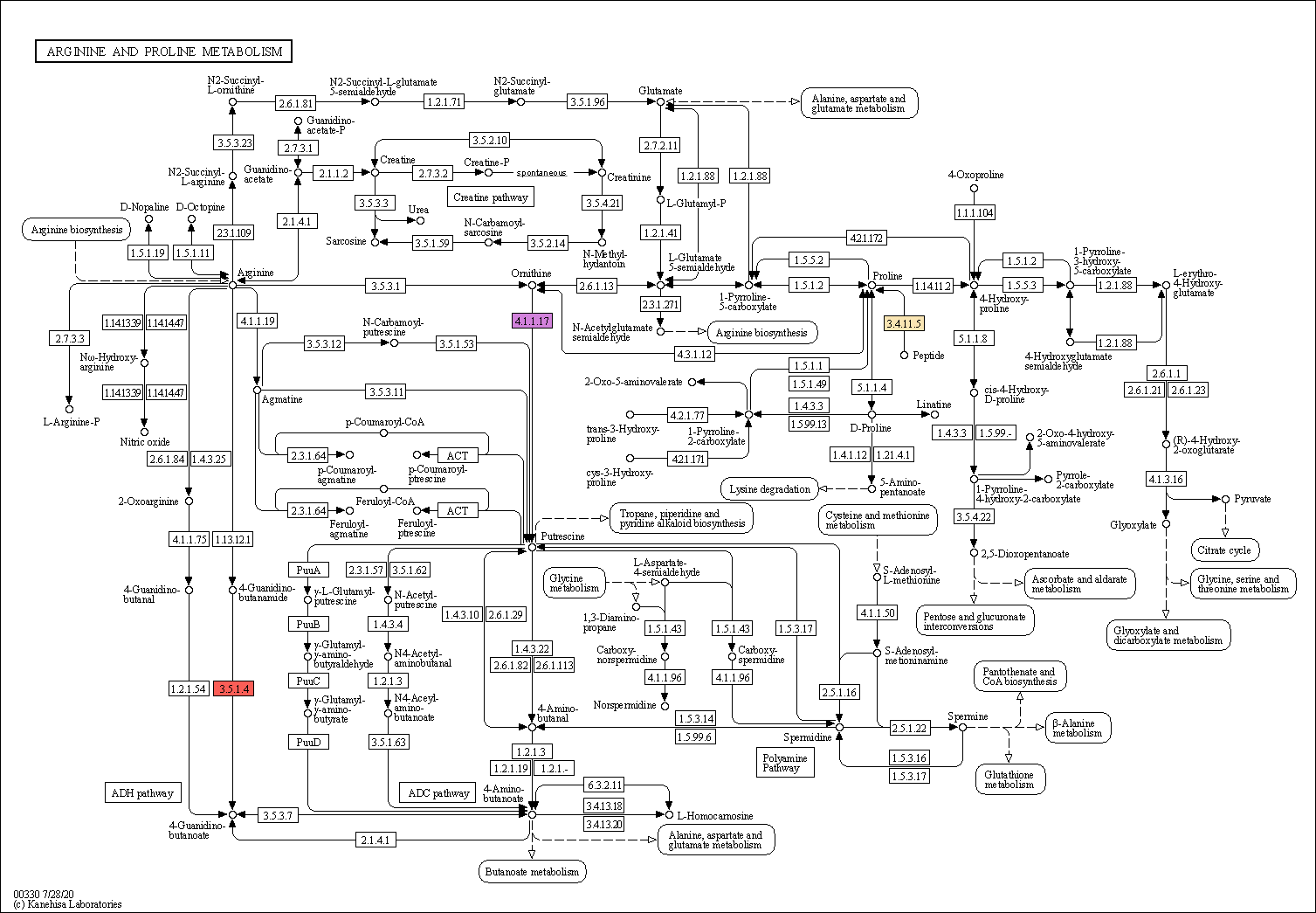

Supplement: Supplementary file 1 [file microorganisms-09-02124-s001.zip › supplementary/Figure S2/Figure S2/KEGG_Patwhays maps_putida consortia vs. pure/high_kegg_map00330_arginine_and_proline_metabolism_20200910_142925.png]

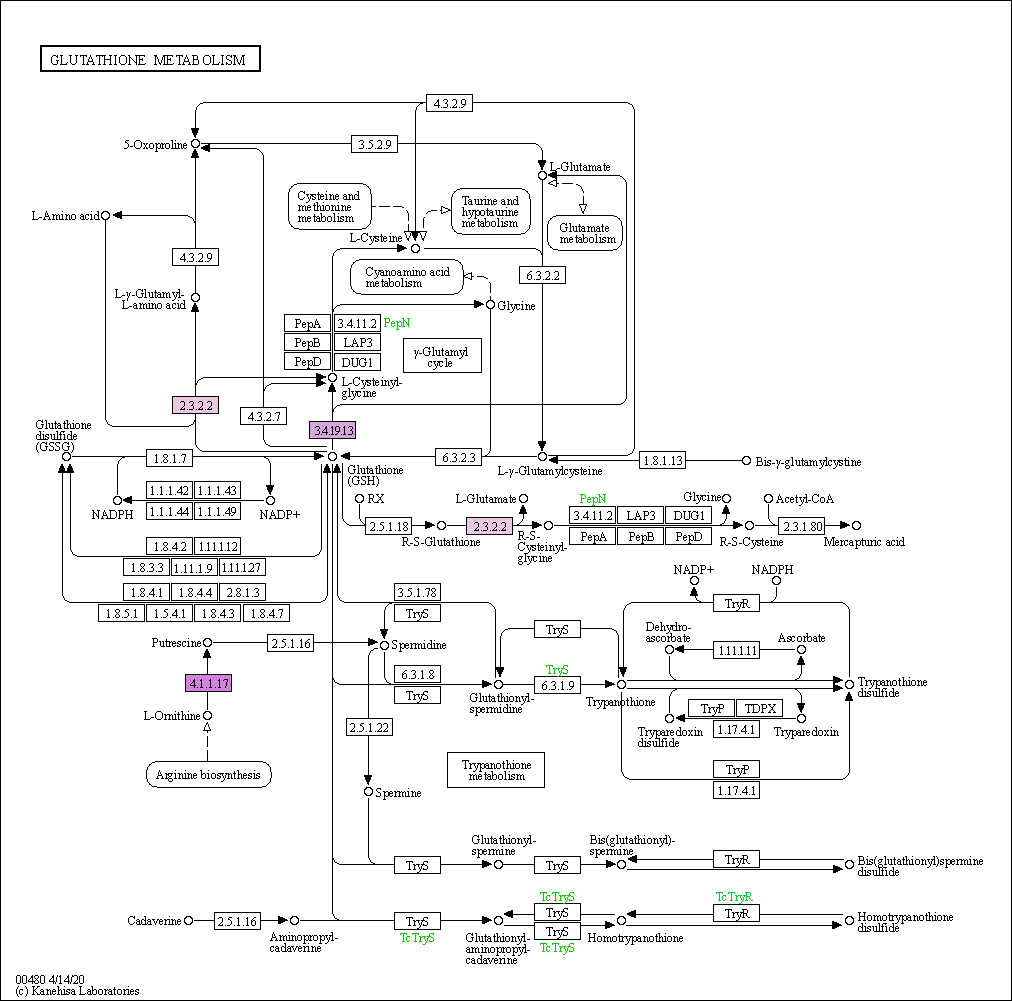

Supplement: Supplementary file 1 [file microorganisms-09-02124-s001.zip › supplementary/Figure S2/Figure S2/KEGG_Patwhays maps_putida consortia vs. pure/high_kegg_map00480_glutathione_metabolism_20200910_142910.png]

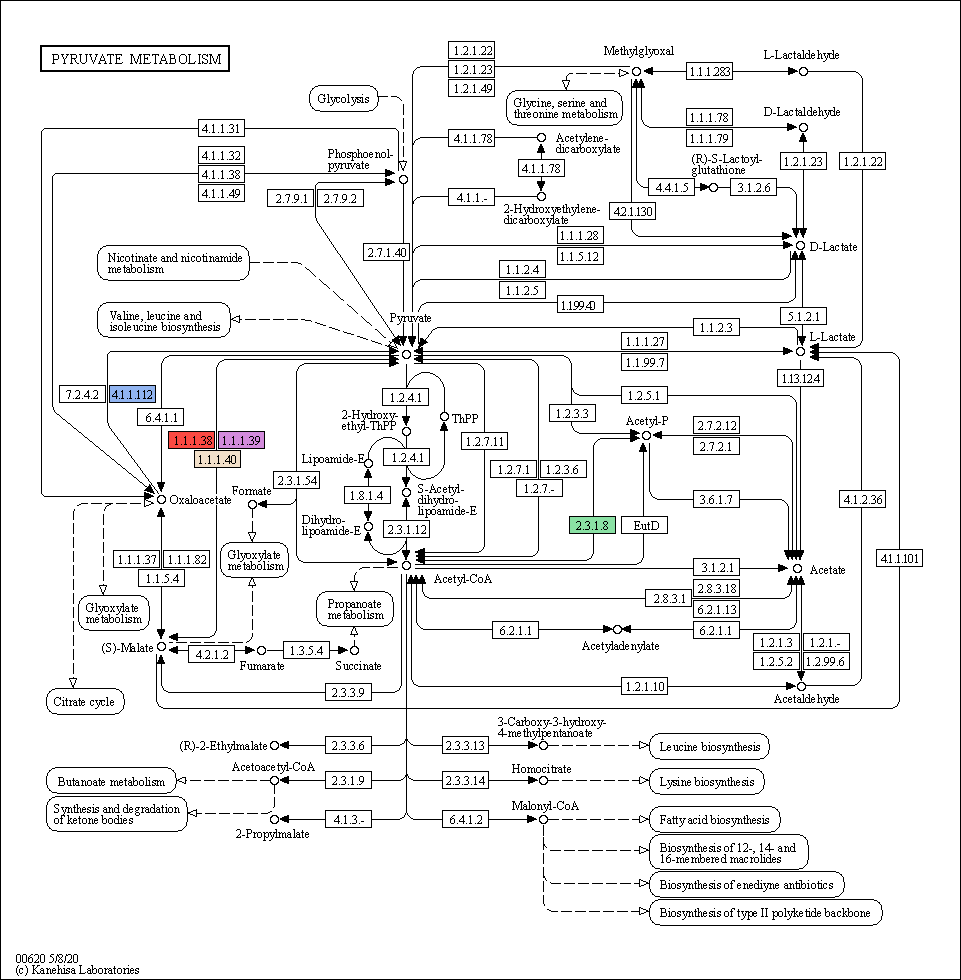

Supplement: Supplementary file 1 [file microorganisms-09-02124-s001.zip › supplementary/Figure S2/Figure S2/KEGG_Patwhays maps_putida consortia vs. pure/high_kegg_map00620_pyruvate_metabolism_20200910_142910.png]

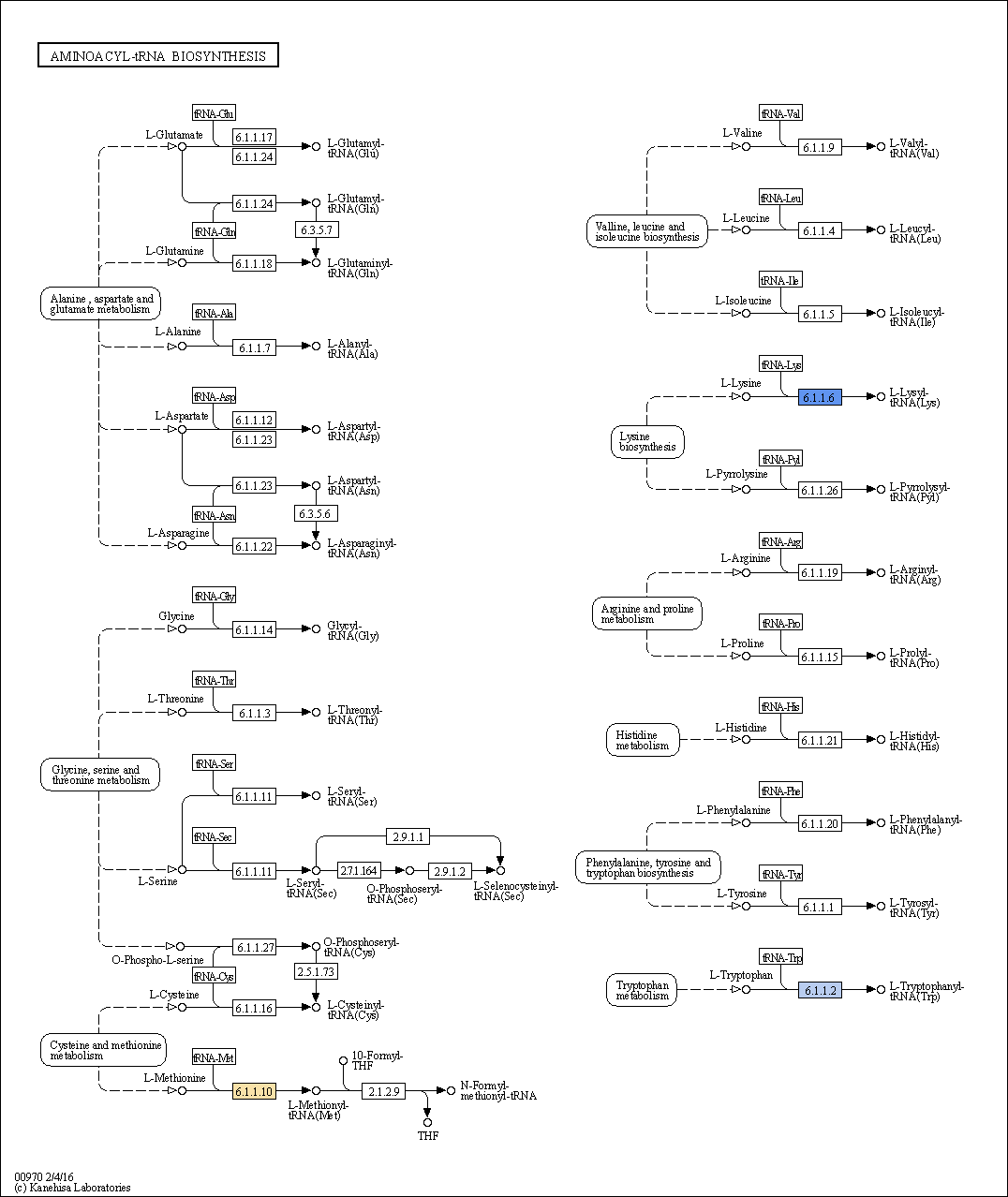

Supplement: Supplementary file 1 [file microorganisms-09-02124-s001.zip › supplementary/Figure S2/Figure S2/KEGG_Patwhays maps_putida consortia vs. pure/high_kegg_map00970_aminoacyl_trna_biosynthesis_20200910_142918.png]

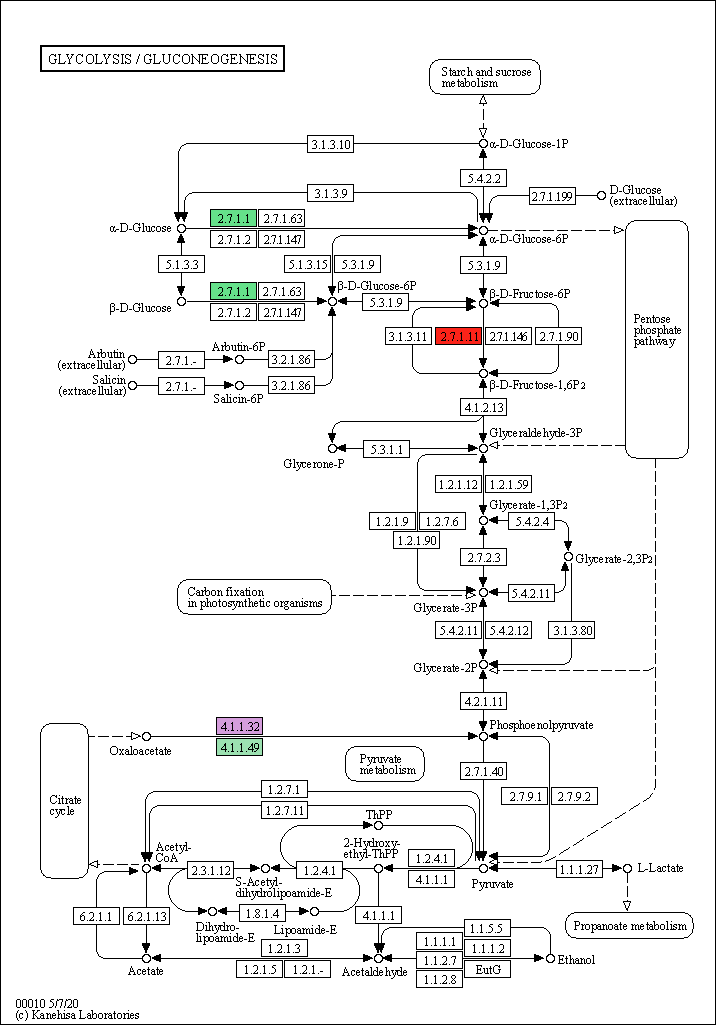

Supplement: Supplementary file 1 [file microorganisms-09-02124-s001.zip › supplementary/Figure S2/Figure S2/KEGG_Patwhays maps_putida consortia vs. pure/low_kegg_map00010_glycolysis_gluconeogenesis_20200910_142758.png]

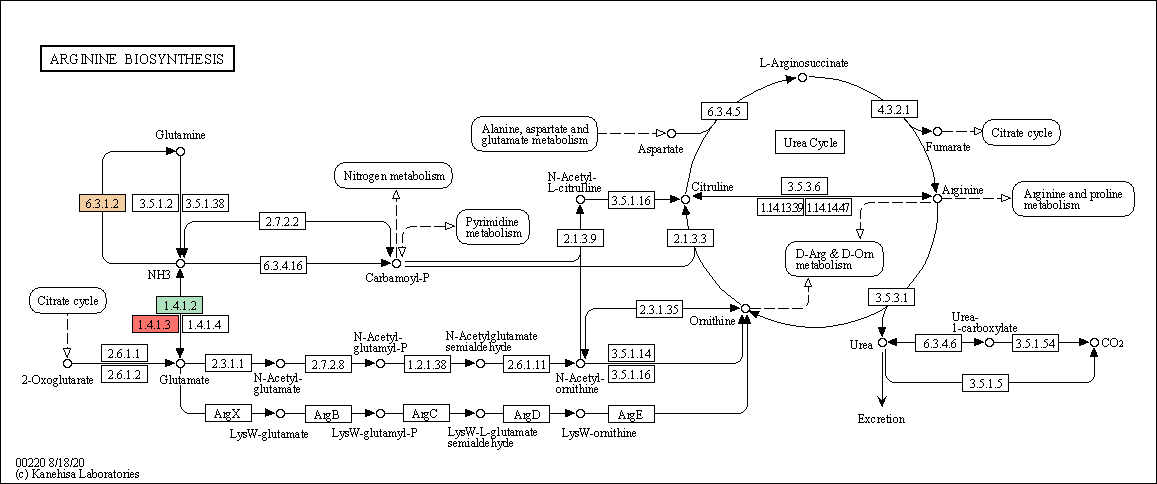

Supplement: Supplementary file 1 [file microorganisms-09-02124-s001.zip › supplementary/Figure S2/Figure S2/KEGG_Patwhays maps_putida consortia vs. pure/low_kegg_map00220_arginine_biosynthesis_20200910_142801.png]

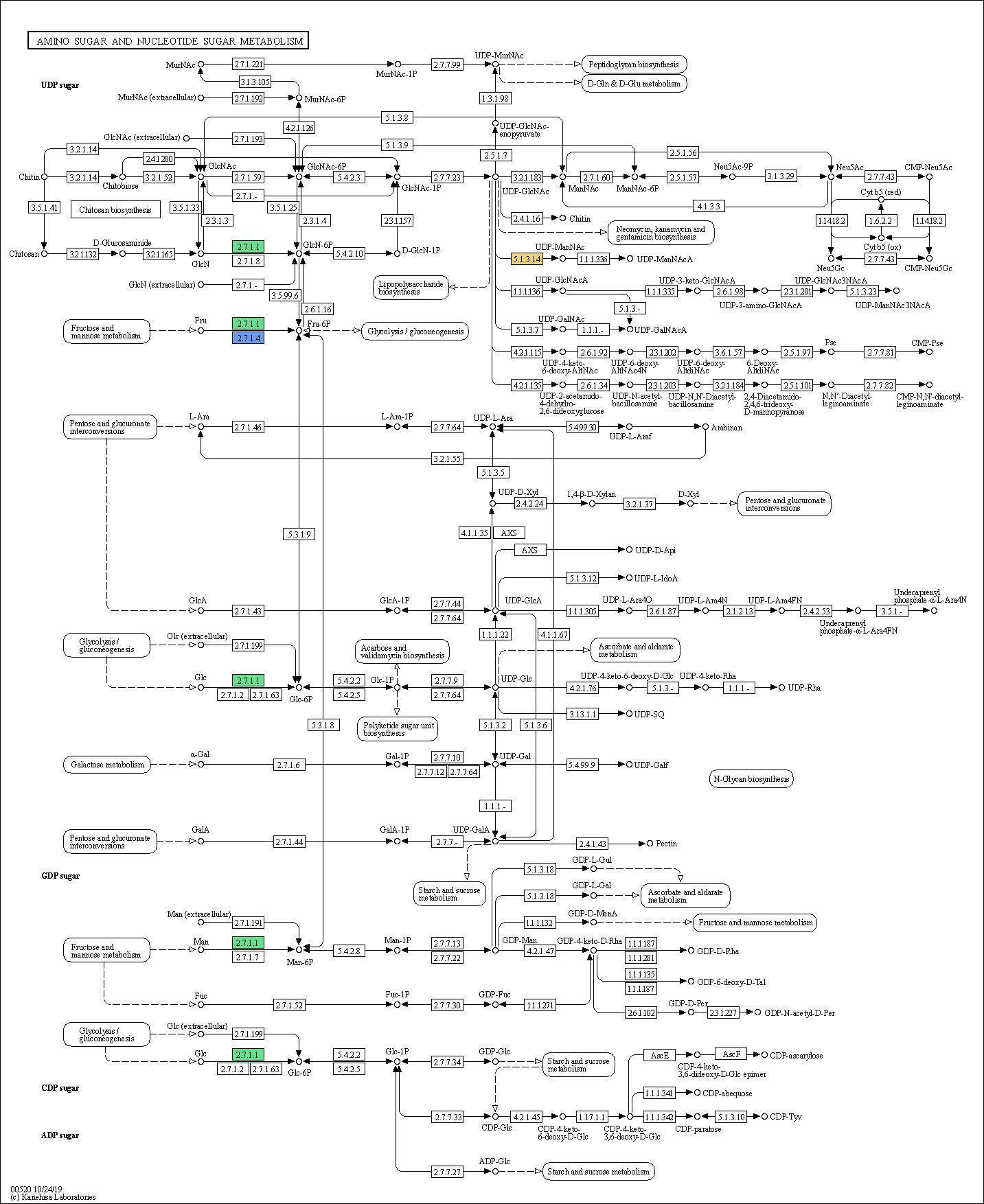

Supplement: Supplementary file 1 [file microorganisms-09-02124-s001.zip › supplementary/Figure S2/Figure S2/KEGG_Patwhays maps_putida consortia vs. pure/low_kegg_map00520_amino_sugar_and_nucleotide_sugar_metabolism_20200910_142802.png]

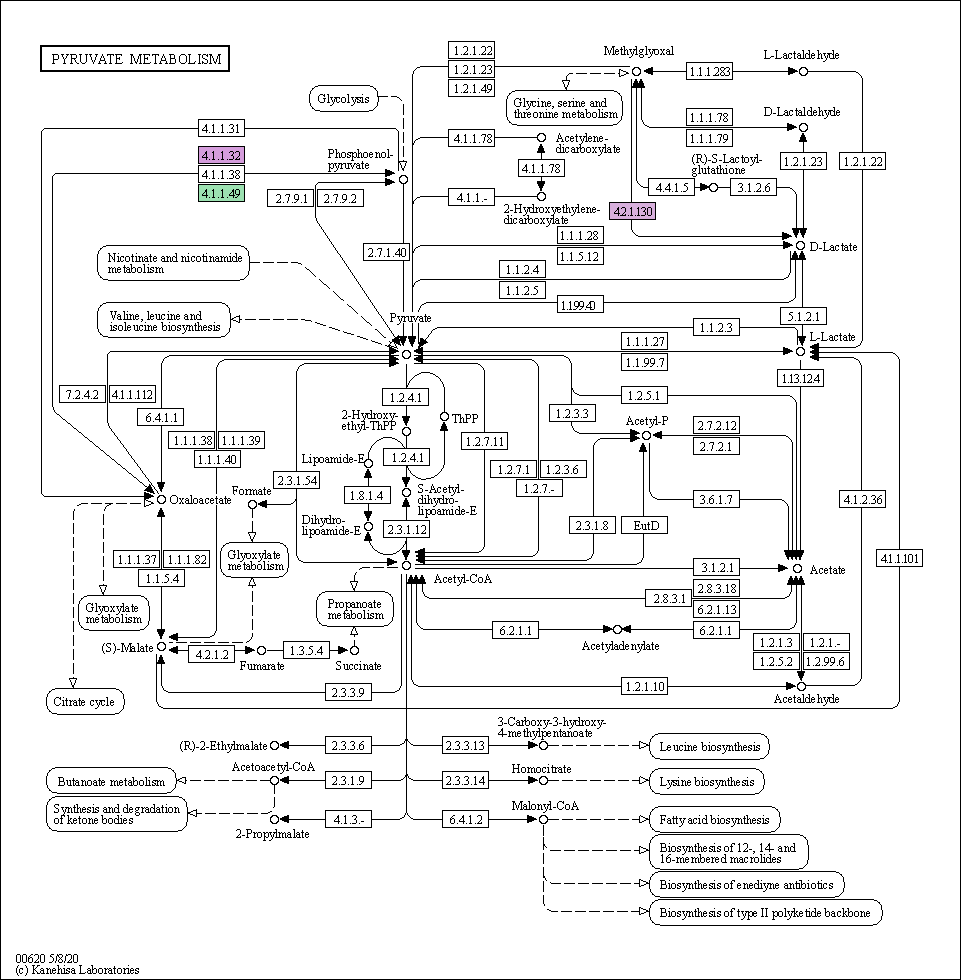

Supplement: Supplementary file 1 [file microorganisms-09-02124-s001.zip › supplementary/Figure S2/Figure S2/KEGG_Patwhays maps_putida consortia vs. pure/low_kegg_map00620_pyruvate_metabolism_20200910_142758.png]

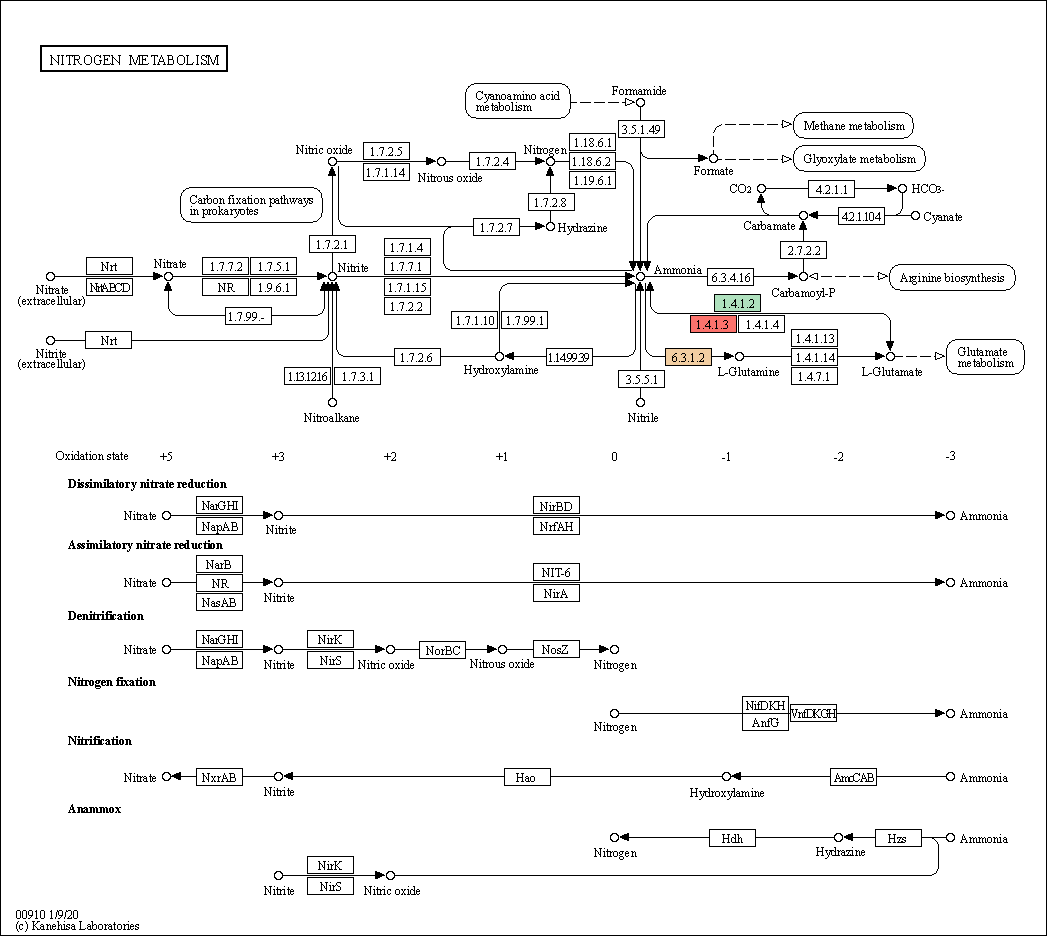

Supplement: Supplementary file 1 [file microorganisms-09-02124-s001.zip › supplementary/Figure S2/Figure S2/KEGG_Patwhays maps_putida consortia vs. pure/low_kegg_map00910_nitrogen_metabolism_20200910_142804.png]
